# Factors affecting long-term efficacy of T regulatory cell-based therapy in type 1 diabetes

**DOI:** 10.1186/s12967-016-1090-7

**Published:** 2016-12-01

**Authors:** Natalia Marek-Trzonkowska, Małgorzata Myśliwiec, Dorota Iwaszkiewicz-Grześ, Mateusz Gliwiński, Ilona Derkowska, Magdalena Żalińska, Maciej Zieliński, Marcelina Grabowska, Hanna Zielińska, Karolina Piekarska, Anna Jaźwińska-Curyłło, Radosław Owczuk, Agnieszka Szadkowska, Krystyna Wyka, Piotr Witkowski, Wojciech Młynarski, Przemysława Jarosz-Chobot, Artur Bossowski, Janusz Siebert, Piotr Trzonkowski

**Affiliations:** 1 Laboratory of Immunoregulation and Cellular Therapies, Department of Family Medicine, Medical University of Gdańsk, Debinki 2, 80-210 Gdańsk, Poland; 2 Department of Pediatric Diabetology and Endocrinology, Medical University of Gdańsk, Debinki 7, 80-210 Gdańsk, Poland; 3 Department of Clinical Immunology and Transplantology, Medical University of Gdańsk, Debinki 7, 80-210 Gdańsk, Poland; 4 Regional Center of Blood Donation and Treatment, Hoene-Wrońskiego 4, 80-210 Gdańsk, Poland; 5 Department of Anaesthesiology and Critical Care, Medical University of Gdańsk, Debinki 7, 80-210 Gdańsk, Poland; 6 Department of Paediatrics, Oncology, Haematology and Diabetology, Medical University of Lodz, Sporna 36/50, 91-738 Lodz, Poland; 7 Section of Transplantation, Department of Surgery, The University of Chicago, 5841 S. Maryland Ave. MC5027, Chicago, IL 60637 USA; 8 Department of Paediatrics, Endocrinology and Diabetes, Medical University of Silesia, Poniatowskiego 15, 40-055 Katowice, Poland; 9 Department of Peadiatrics, Endocrinology, Diabetology with Cardiology Division, Medical University of Bialystok, Jana Kilińskiego 1, 15-089 Białystok, Poland

**Keywords:** Diabetes type 1, Children, T regulatory cells, Immunotherapy

## Abstract

**Background:**

Recent studies suggest that immunotherapy using T regulatory cells (Tregs) prolongs remission in type 1 diabetes (T1DM). Here, we report factors that possibly affect the efficacy of this treatment.

**Methods:**

The metabolic and immune background of 12 children with recently diagnosed T1DM, as well as that of untreated subjects, during a 2-year follow-up is presented. Patients were treated with up to 30 × 10^6^/kg b.w. of autologous expanded CD3^+^CD4^+^CD25^high^CD127^−^ Tregs.

**Results:**

The disease progressed and all patients were insulin-dependent 2 years after inclusion. The β-cell function measured by c-peptide levels and the use of insulin were the best preserved in patients treated with two doses of Tregs (3/6 in remission), less so after one dose (1/6 in remission) and the worst in untreated controls (no remissions). Increased levels of Tregs could be seen in peripheral blood after their adoptive transfer together with the shift from naïve CD62L^+^CD45RA^+^ to memory CD62L^+^CD45RA^−^ Tregs. Increasing serum levels of proinflammatory cytokines were found: IL6 increased in all subjects, while IL1 and TNFα increased only in untreated group. Therapeutic Tregs were dependent on IL2, and their survival could be improved by other lymphocytes.

**Conclusions:**

The disease progression was associated with changing proportions of naïve and memory Tregs and slowly increasing proinflammatory activity, which was only partially controlled by the administered Tregs. The therapeutic cells were highly dependent on IL2. We conclude that the therapy should be administered at the earliest to protect the highest possible mass of islets and also to utilize the preserved content of Tregs in the earlier phases of T1DM.

*Trial registration* http://www.controlled-trials.com/ISRCTN06128462; registered retrospectively

**Electronic supplementary material:**

The online version of this article (doi:10.1186/s12967-016-1090-7) contains supplementary material, which is available to authorized users.

## Background

Type 1 diabetes (T1DM) is an emerging medical problem, since there is no causal treatment and patients inevitably develop full onset of the disease, e.g., in Poland the consequent morbidity doubles every 10 years [1]. The majority of patients are children and the initial manifestation can often be severe, including deep ketoacidosis or coma. It is, therefore, important to investigate novel treatments, aiming at early intervention, while a significant mass of β-cells is still present and can be preserved.

A common consensus exists that the disease develops as a result of the attack of autoaggressive T-cells that infiltrate pancreatic islets and destroy insulin-producing β-cells [2]. This autoaggression is usually unleashed when suppressive subsets, such as CD3^+^CD4^+^FoxP3^+^ T regulatory cells (Tregs), are somehow impaired [3]. Indeed, the adoptive transfer of Tregs was confirmed in animal models as an effective way to stop or delay the progression of the disease [4]. Translational studies in humans seem to confirm this observation, however, the disease still progresses in patients treated with Tregs preparation. It is, therefore, necessary to identify the factors that influence the efficacy of this therapy in the clinical setting.

Starting from 2009, therapy using T regulatory cells moved to the clinical stage and its efficacy is currently being assessed in various conditions, including T1DM [5]. Our group performed several such studies, including that for the treatment of T1DM [5–8]. In this paper, apart from the clinical background, we will present some immunity studies in order to identify the factors that possibly influence the efficacy of the adoptive transfer of Tregs in T1DM.

## Methods

### Protocol and treatment

This was an open-labeled study conducted according to the Declaration of Helsinki principles and was approved by the Ethics Committee of the Medical University of Gdańsk, Poland (NKEBN/8/2010 with amendments). The trial was registered at the Current Controlled Trials database: http://www.controlled-trials.com/ISRCTN06128462 (Additional file 1). Written informed consent was received from parents of all the participants and from the patients themselves, if above 16-years of age.

As described in earlier reports [7, 8], 12 Caucasian children from the Polish population with recently diagnosed T1DM were treated with ex vivo expanded autologous Tregs. The general health and metabolic status of the treated individuals were followed for 24 months after inclusion to the study along with those of ten untreated, control patients matched for age, sex and disease duration. The main inclusion criteria were: having autoimmune T1DM diagnosed within 2 months; the presence of at least one type of anti-islet autoantibody anti-GAD, anti-IA2, IAA, or ICA; age–5 to 18 years; fasting plasma C-peptide levels >0.4 ng/mL and proper management of diabetes. The control group was recruited among patients who fulfilled the same criteria, but did not qualify for admission to the treated group due to inadequate venous access (Table 1).

**Table 1:** Clinical characteristics of the patients

	Treated (n = 12)	Not treated (n = 10)
Age (years) [median; min–max]	12.2; 8–16	11.5; 7–16
BMI [median; min–max]	17.1; 12.5–23.5	16.7; 14.2–20.8
Polydipsia at diagnosis (number of patients)	5	8
Polyuria at diagnosis (number of patients)	5	3
Loss of weight at diagnosis (number of patients)	4	3
pH at diagnosis (capillary blood) [median; min–max]	7.40; 7.36–7.42	7.39; 7.35–7.53
pO_2_ at diagnosis (capillary blood—mmHg) [median; min–max]	69.3; 24.1–88.0	69.5; 56.0–86.6
pCO_2_ at diagnosis (capillary blood—mmHg) [median; min–max]	39.6; 28.0–46.9	38.0; 24.0–40.7
HCO_3_ at diagnosis (capillary blood—mmHg) [median; min–max]	24.15; 18.8–27.4	23.6; 21.3–25.2
Acid/base balance at diagnosis (BE—mEq/l) [median; min–max]	0.05; −7.8–3.2	−0.5; −3.8–0.9
Sat02 at diagnosis (capillary blood—%) [median; min–max]	94.1; 90.2–97.3	95.4; 92.4–97.2
Anti-GAD65 antibody (number of patients)	9	9
ICA (number of patients)	7	5
IAA (number of patients)	7	4

Tregs were isolated from the patients’ peripheral blood with a GMP-compliant FACS sorter (Influx; BDBiosciences, USA). The purity of Tregs after sorting was ≈98% (range 97–100%). The expansion was performed under GMP conditions and according to our previously described protocol using anti-CD3/anti-CD28 beads, interleukin 2 (IL-2) and autologous serum. The final product on release kept the FoxP3 expression above 90% [median (min.–max) = 91% (90–97)] [9].

The dose-escalation scheme of Tregs administration was: 10 × 10^6^ of Tregs/kg b.w. in a single infusion (three patients), 20 × 10^6^ of Tregs/kg b.w. in a single infusion (three patients), and a total of 30 × 10^6^ of Tregs/kg b.w. in two infusions (six patients), with the second dose being administered 6–9 months after the first one. Two patients were lost to follow-up at +6 and +9 months, while ten patients completed the trial.

The primary endpoints of the trial were safety and remission defined as daily dose of insulin (DDI) ≤0.5 UI/kg b.w. and fasting C-peptide levels >0.5 ng/mL 1 year after recruitment. Secondary endpoints included the immune background of the patients treated with the preparation of Tregs.

### Metabolic and immune responses

Fasting C-peptide levels, fasting glucose, HbA1c and T1DM autoantibody [glutamic acid decarboxylase autoantibody (anti-GAD65), insulin autoantibody (IAA), insulin antigen 2 antibody (IA2) and zinc transporter 8 autoantibody (anti-ZnT8)] levels were measured during a 24-month-long follow-up at different time points, as previously described [7, 8]. The mixed meal tolerance test (MMTT) was performed according to standard criteria on the day of the 24th month of follow-up [10].

Immune phenotyping was performed using a seven-color panel: CD3/CD4/CD25/CD127/CD45RA/CD62L/FoxP3. Two phenotypes of Tregs were analyzed: CD3^+^CD4^+^FoxP3^+^ and CD3^+^CD4^+^CD25^high^CD127^−^ T-cells. The gate CD25^high^CD127^−^ from the later population was also used to assess the content of FoxP3^+^ T-cells in order to estimate an overlap between the two phenotypes of Tregs. Finally, CD3^+^CD4^+^FoxP3^+^ T-cells were subdivided into naive CD3^+^CD4^+^FoxP3^+^CD62L^+^CD45RA^+^ (Tn) Tregs, central memory, CD3^+^CD4^+^FoxP3^+^CD62L^+^CD45RA^−^ (Tcm) Tregs, and effector memory, CD3^+^CD4^+^FoxP3^+^CD62L^−^CD45RA^−^ (Tem) Tregs [9].

The following anti-human monoclonal antibodies were used in this procedure (fluorochrome/class/clone): anti-CD3 (PacificBlue/IgG1/UCHT1), anti-CD4 (PerCP/IgG1/RPA-T4), anti-CD25 (PE/IgG1/M-A251), anti-CD127 (FITC/IgG1/hIL-7R-M21) and, anti-CD45RA (PE-Cy7/IgG1/L48). All of the antibodies were purchased from BDBiosciences, Poland. Anti-CD62L (APC-Cy7/IgG1/3B5) was supplied by Invitrogen, USA, and the FoxP3 staining kit by eBioscience, USA.

Serum levels of IFNγ, VEGF, TNFα, IL1, IL2, IL4, IL6, IL8, IL10 and, IL12 were measured with the Luminex Bead based Multiplex Assay (ebioscience, USA), while BAFF, TGFβ, and, IL17 were measured with Quantikine High Sensitivity ELISA kit (R&D Systems, USA). All assays were performed according to the manufacturers’ instructions.

### IL2 dependency tests

Samples of cells from Tregs expansions administered to the patients and autologous CD3^+^CD4^+^CD127^+^ conventional/effector T-cells (Teffs) expanded along with Tregs were cultured for additional 8 days after the release of Treg products to the clinic. Tregs and Teffs were cultured separately or co-cultured in a 1:1 ratio, 1 × 10^6^ cells/well in 24-well plates, in a 5% CO_2_ atmosphere, at 37.0 °C, in the following concentrations of IL2: 0.0, 10.0, 100.0 and 1000 UI/mL. Survival of the cells was measured every day by flow cytometry using 7-aminoactinomycin D staining (7-AAD, Via-probe BDBiosciences, Poland).

### Statistical analysis

Data were computed with the software Statistica 10.0 (Statsoft, Poland). As indicated by the distribution of the variables non-parametric tests were used. The analysis was performed using Kruskal–Wallis ANOVA (KW), the U-Mann–Whitney test (MW), Wilcoxon test, and Spearman’s rank correlation. The p value was considered statistically significant when <0.05 (Additional file 2).

## Results

### β-cell function

β-cell function was best preserved in patients treated with two doses of Tregs, slightly less preserved in those treated with one dose and the least preserved in untreated controls. The criteria of remission were achieved at the 24th month of follow-up in 3 out of 6 patients treated with two doses of Tregs, 1 out of 6 patients treated with a single dose of Tregs, while no remission occurred in the control group. There were no additional adverse effects, as compared to previous reports [7, 8].

**Fig. 1:**
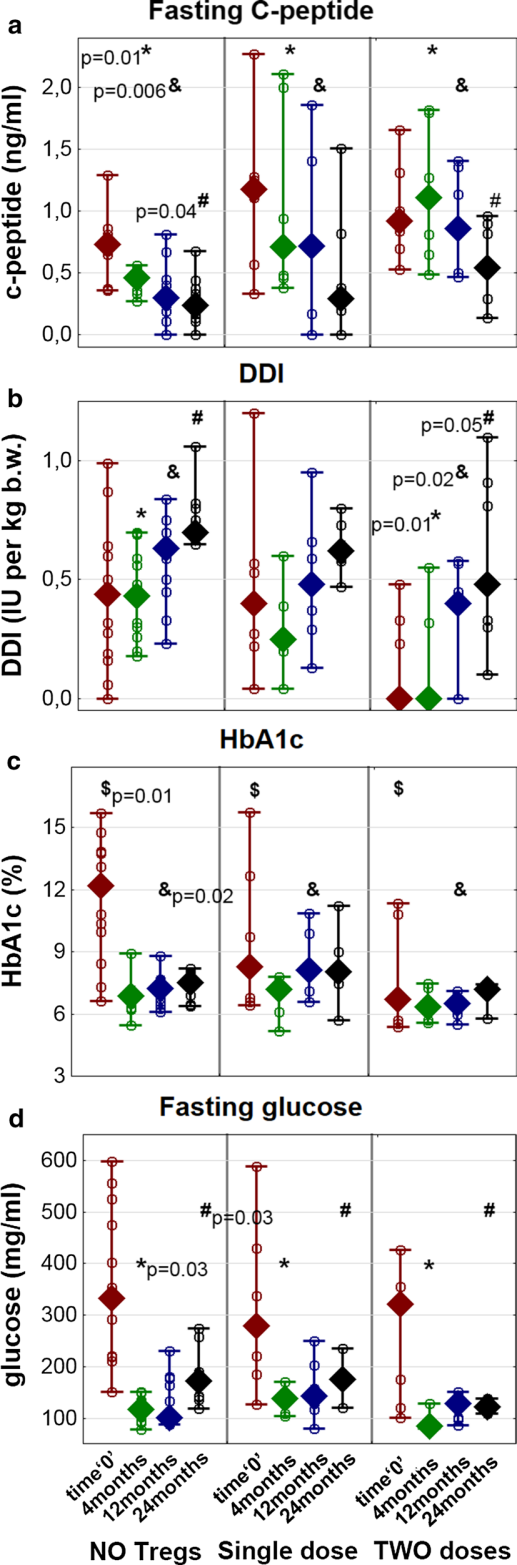
Metabolic markers of T1DM in studied groups. The figure depicts levels of fasting C-peptide (**a**), daily doses of insulin/kg b.w. (DDI/kg) (**b**), levels of HbA1c (**c**) and fasting glucose (**d**) in T1DM children during the follow-up: NO Tregs—untreated control group, single dose—patients treated with a single infusion, and two doses—patients treated with two infusions of Tregs. The following values of HbA1c in NGSP units (%) match the IFCC units (mmol/mol): 3 = 9, 6 = 42, 9 = 75, 12 = 108, and 15 = 140. Data are presented as medians (min.–max.), while *open circles* represent particular patients. The significant differences in p values are shown in the figure: **a** comparisons of C-peptide levels in Kruskal–Wallis ANOVA between three groups at the 4th month (*) and 12 month (&) and Mann–Whitney U test comparison between the untreated group and the group treated with two doses of Tregs at the 24th month (#); **b** comparisons of daily doses of insulin in Kruskal–Wallis ANOVA between three groups at the 4th (*), 12 (&), and 24th month (#); **c** comparisons of hemoglobin A1c levels in Kruskal–Wallis ANOVA between three groups at day 0 ($) and the 12 month (&); **d** comparisons of fasting glucose levels in Kruskal–Wallis ANOVA between three groups at the 4th month (*) and 24th month (#)

As compared to untreated controls, the fasting C-peptide level was significantly higher in individuals treated with a single or two doses of Tregs throughout the study (KW p < 0.05 on 4th and 12 month of follow-up). This difference between the single dose group and the untreated controls became insignificant at 2 years’ post-recruitment (MW p = 0.43); however, it was still significant when comparing the two dose and the untreated controls (MW p = 0.04) (Fig. 1).

A significant difference was also seen when daily insulin requirements were analyzed. When compared to untreated controls, both groups treated with Tregs required significantly lower doses of insulin throughout the study (KW p < 0.05). The group treated with two Tregs doses required the lowest doses of exogenous insulin, which maintained the levels of metabolic parameters, such as HbA1c and fasting glucose under control, notably at 2 years post inclusion (KW p < 0.05 at the 4th and the 24 month of follow-up for fasting glucose). Importantly, two patients in the group treated with two doses were insulin independent more than 1 year after infusion.

**Fig. 2:**
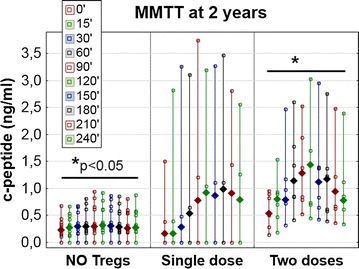
Mixed meal tolerance test performed at the 24th month of follow-up. Levels of C-peptide in MMTT in T1DM children at the 24th month of follow-up are displayed separately for untreated controls (NO tregs), patients treated with single infusion of Tregs (single dose) and patients treated with two infusions of Tregs (two doses). The values are given as medians (min.–max.) and the legend shows the timing of C-peptide measurements in minutes during the test. The *horizontal lines* represent significant differences (p < 0.05) in C-peptide levels in Kruskal–Wallis ANOVA between the untreated group and the group treated with two doses of Tregs in all ten time points of the test

The improved function of the islets in the groups treated with Tregs was also confirmed with mixed meal tolerance test (MMTT) at 2 years’ post-inclusion. Both treated groups were characterized by improved stimulated C-peptide profiles compared to those of the untreated group. The difference between the group treated with two doses and the untreated group was significant at all time points after the stimulation (KW p < 0.05), whereas the difference between the group treated with one dose and the untreated group was insignificant (KW p > 0.05) (Fig. 2).

### Tregs levels in vivo

**Fig. 3:**
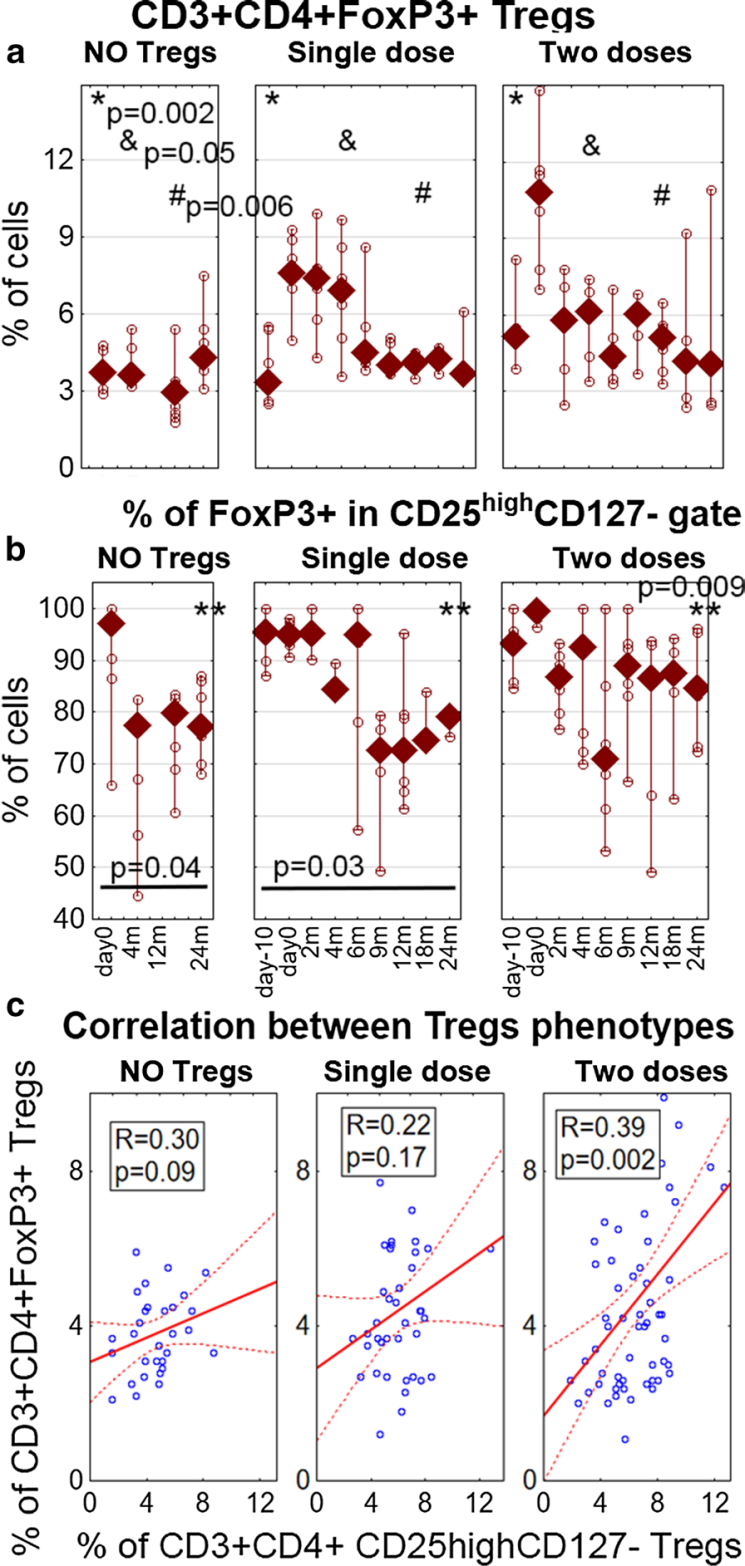
The levels of different phenotypes of Tregs and their correlation. **a** The percentage of Tregs measured as CD3+ CD4+ FoxP3+ cells during the follow-up. The p values show the significance in Kruskal–Wallis ANOVA comparisons between three groups at day 0 (*), 4th (&) and 12 month (#). **b** The percentage of FoxP3+ cells measured in the CD3+ CD4+ CD25highCD127− gate. The p values and the lines represent the significance of Wilcoxon test comparisons: day 0 vs. 24th month in particular groups and **p represents the significance of Kruskal–Wallis ANOVA comparison between three groups at the 24th month. The values for untreated controls (NO Tregs), patients treated with a single infusion (single dose), and patients treated with two infusions (two doses) of Tregs are presented as medians (min.–max.); the *open circles* represent values from particular patients. **c** Spearman’s rank correlation between CD3+ CD4+ FoxP3+ and CD3+ CD4+ CD25highCD127-phenotypes used to assess the percentage of Tregs in the follow-up (R and p are given for particular groups)

The level of Tregs increased each time the cells were administered (KW p > 0.05) (Fig. 3a). Nevertheless, the increased level of Tregs was not sustained; it decreased to the baseline at 2 years after the first infusion (KW p < 0.05). In addition, the increase in the level of Tregs after the second dose was not as high as after the first administration. Although there was a correlation between the percentages of the two phenotypes of Tregs CD3^+^CD4^+^FoxP3^+^ and CD3^+^CD4^+^CD25^high^CD127^−^, this was significant throughout the follow-up only in the group receiving two doses of Tregs (Fig. 3b, c). The analysis of FoxP3^+^ cells in the CD25^high^CD127^−^ gate revealed that the percentage of FoxP3^+^ cells was significantly decreasing in this gate in untreated patients and in those receiving a single dose of Tregs as T1DM progressed (Fig. 3b). On the other hand, there was no significant decrease in the percentage of FoxP3^+^ cells in the CD3^+^CD4^+^CD25^high^CD127^−^ gate in patients treated with two doses of Tregs. Among the three groups analyzed, this percentage was highest in the group treated with two doses of Tregs at the 24th month of follow-up (Kruskal–Wallis ANOVA χ^2^ = 6.66 p = 0.009).

### Tregs subsets in vivo

**Fig. 4:**
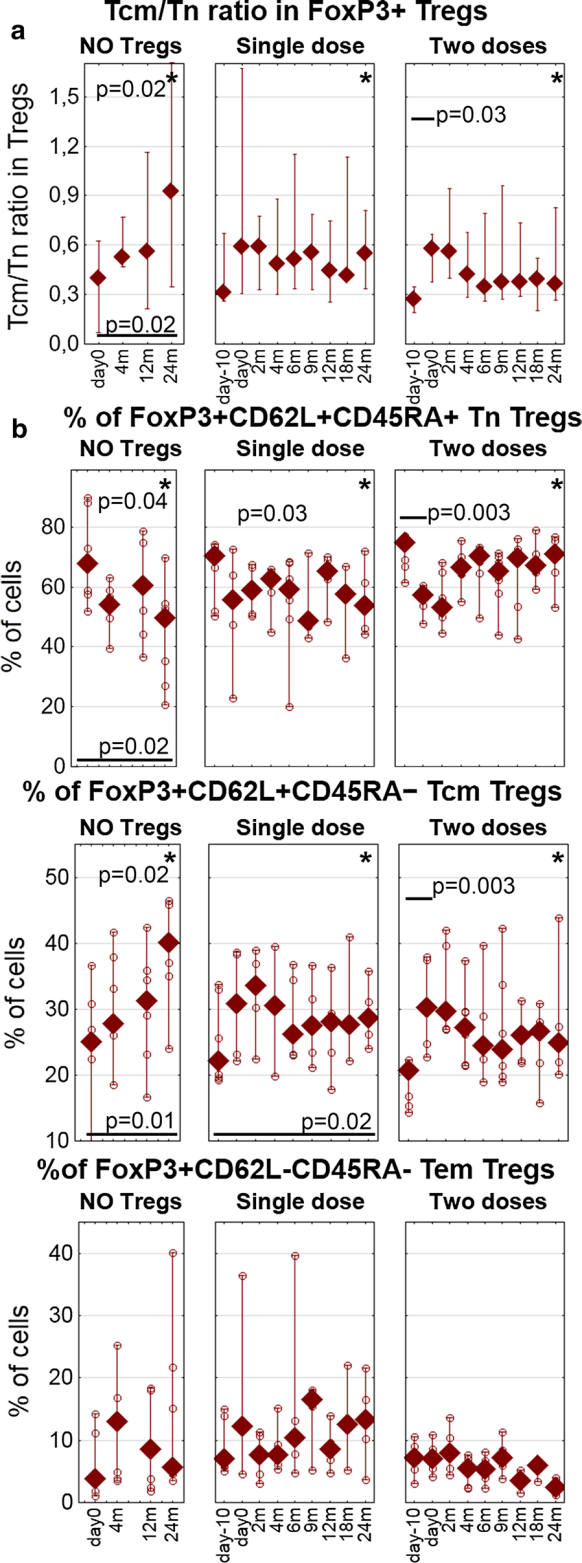
Subsets of tregs in the follow-up. **a** The ratio between Tcm and Tn CD3+ CD4+ FoxP3+ Tregs is shown to better visualize the Tcm/Tn swap. **b** The percentages of Tn Tregs (*upper panel*), Tcm Tregs (*middle panel*), and Tem Tregs (*bottom panel*). The values for untreated controls (NO Tregs), patients treated with single infusion (single dose), and patients treated with two infusions (two doses) during the follow-up. Data are presented as medians (min.–max.), and the *open circles* represent particular patients. The *p values represent the significance in Kruskal–Wallis ANOVA comparisons between three groups at the 24th month; p values and *short lines* represent the significance of Wilcoxon test comparisons immediately after Tregs infusions in the treated groups; p values and long lines represent the significance of Wilcoxon test comparisons between day-10 or day 0 vs. the 24th month in particular groups

The percentage of CD62L^+^CD45RA^−^ Tcm Tregs increased after the administration of Tregs. There was a significant shift from the naïve CD62L^+^CD45RA^+^ Tn phenotype to the CD62L^+^CD45RA^−^, Tcm phenotype of Tregs immediately after the first infusion in both treated groups. (Wilcoxon test: first dose p < 0.05, second dose p > 0.05) (Fig. 4).

### Immune markers of disease progression in peripheral blood

The control untreated group was characterized by a significant shift of Treg subsets during the study (Fig. 4). Compared to the baseline, there was an increase in the level of Tcm Tregs at the expense of naïve Tregs at the 24th month of follow-up. Similarly, but not as significant, changes were seen in patients treated with a single dose of Tregs. These differences were the least noticeable in the group treated with two doses of Tregs (Wilcoxon test: no Tregs p < 0.05, treated groups p > 0.05). Among the groups, the patients treated with two doses of Tregs preserved the proportions of Treg subsets at the 24th month, having the highest level of Tn Tregs and the lowest level of Tem Tregs (KW p < 0.05) (Fig. 4b).

**Fig. 5:**
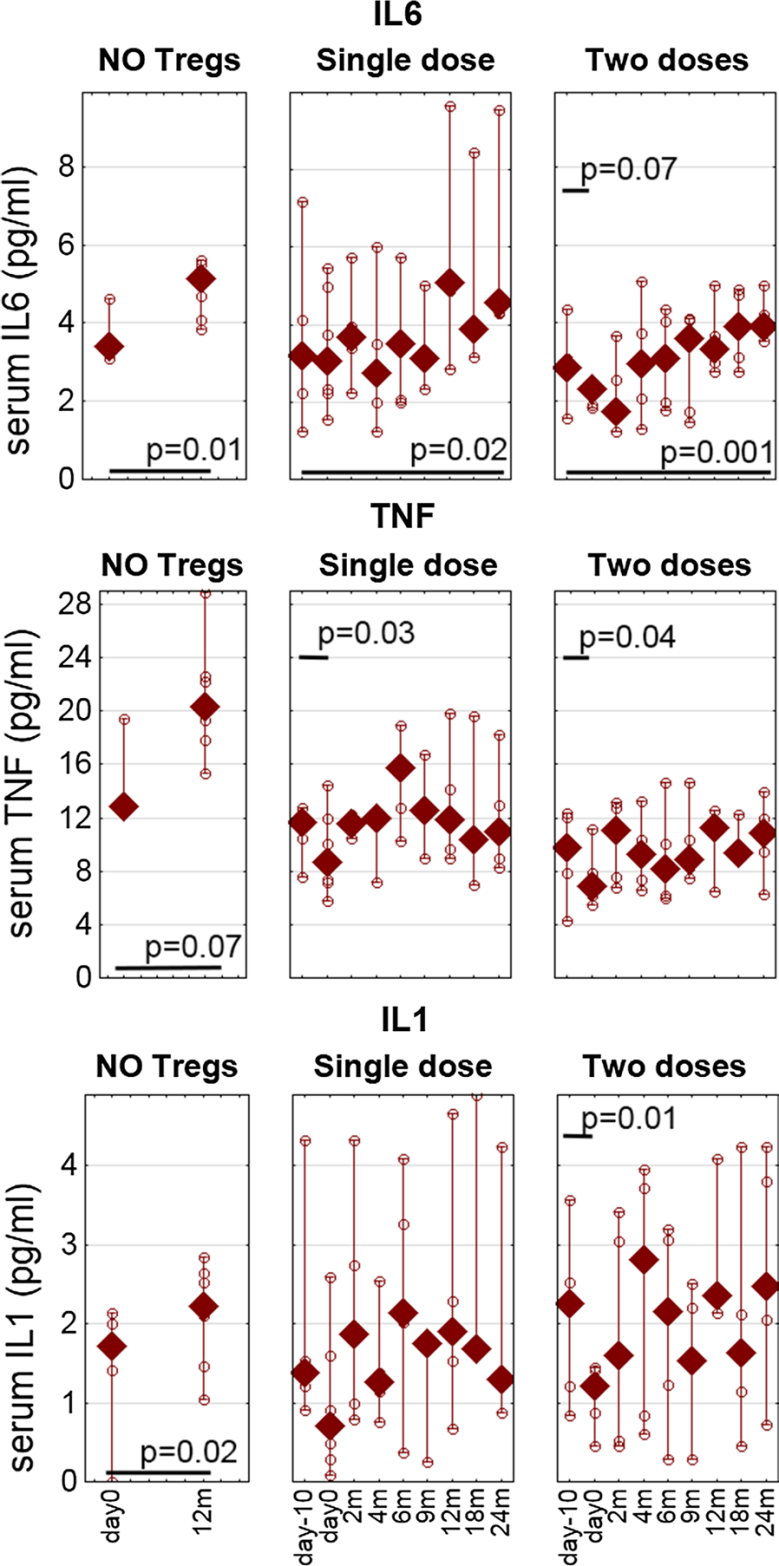
The levels of proinflammatory cytokines in the follow-up. Serum concentrations of IL6 (*upper panel*) TNFα (*middle panel*) and IL1 (*bottom panel*) were measured during the follow-up. The values for untreated controls (NO Tregs), patients treated with a single infusion (single dose) and patients treated with two infusions (two doses) of Tregs are presented as medians (min.–max.), *open circles* represent values from particular patients. The p values and *short lines* represent the significance of Wilcoxon test comparisons immediately after Tregs infusions in treated groups; p values and *long lines* represent the significance of Wilcoxon’ test comparisons between day 0 vs. the 12 month in the untreated group and day-10 vs. the 24th month in treated groups

Among the cytokines measured from sera, proinflammatory ones revealed some pattern during the follow-up (Fig. 5). Serum levels of IL6 consistently increased with time, regardless of the therapy. All patients revealed higher levels of IL6 at the 24th month (Wilcoxon test, day 0 vs. 24 month: p < 0.05). There was a slight and transient decrease immediately after the first dose only in the group administered with two doses of Tregs. Furthermore, the first infusion of Tregs in both treated groups was associated with a temporary decrease in the level of TNFα and IL1 (Wilcoxon test: first dose p < 0.05, second dose p > 0.05).

In the control untreated group, the levels of TNFα, IL1, and IL6 were higher at the 12 month than at the start of the study (Wilcoxon test: p < 0.05). Unfortunately, we have no data for these cytokines from the 24th month of follow-up. Apart from IL2, no significant changes were found in serum levels of other cytokines measured.

**Fig. 6:**
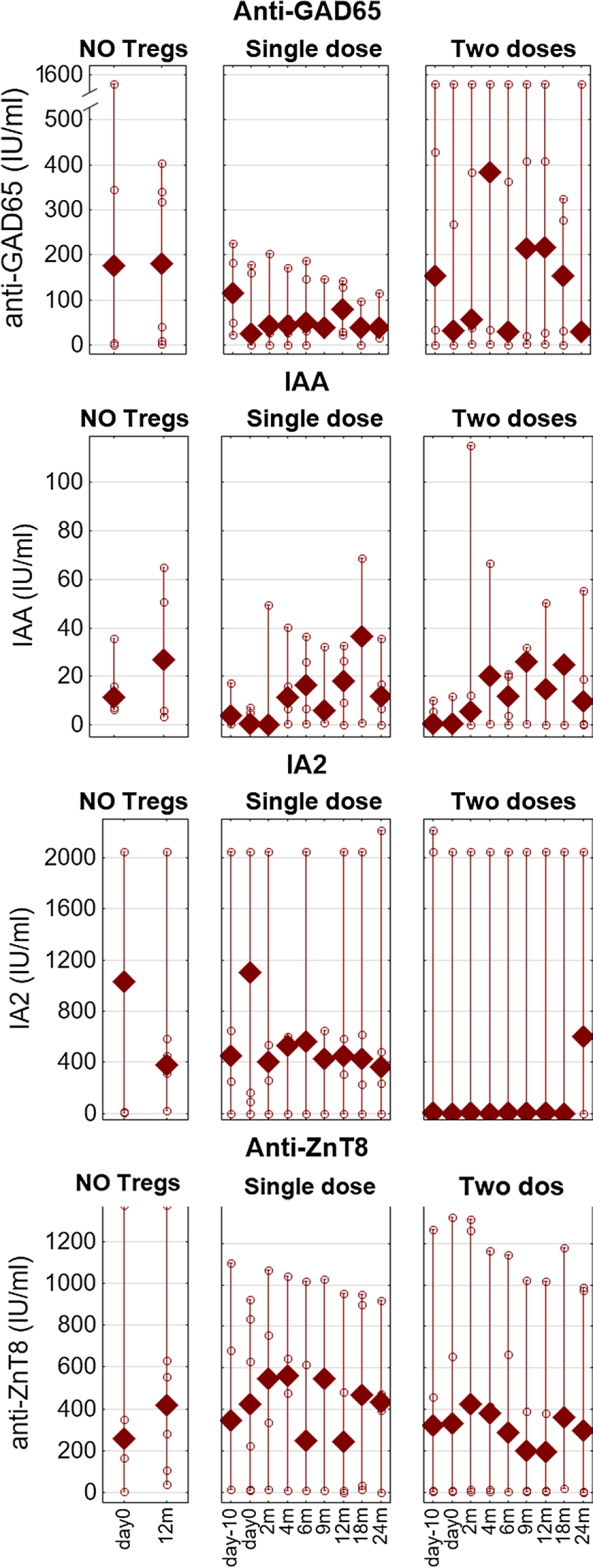
T1DM autoantibodies in the follow-up. The levels of anti-GAD65, IAA, IA2 and anti-ZnT8 autoantibodies were measured. The values are shown for the untreated controls (NO Tregs), patients treated with single infusion (single dose) and patients treated with two infusions (two doses) during the follow-up. Data are presented as medians (min.–max.), and the *open circles* represent particular patients

There was a correlation between the level of CD3^+^CD4^+^FoxP3^+^ Tregs in vivo and IA2 (Spearman’s rank correlation R = −0.304, p = 0.05; Additional file 2: Figure S1). However, this was significant only when all groups were taken together in the analysis. No other T1DM autoantibodies (anti-GAD65, IAA, or anti-ZnT8) correlated with Treg levels, the dose of Tregs administered, or the cytokines assessed (Fig. 6).

### Tregs and IL2

**Fig. 7:**
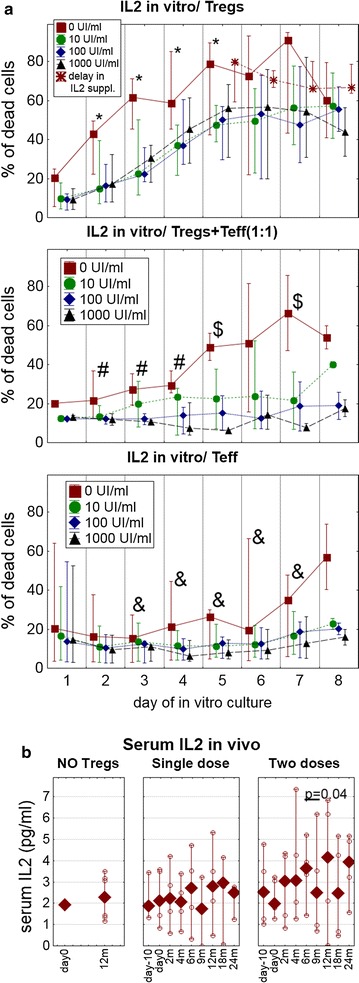
Tregs survival and dependency on IL2. **a** Survival of expanded Tregs was assessed in vitro after release from GMP laboratory for further 8 more days in various concentrations of IL2: 0.0, 10.0, 100.0 and 1000 UI/mL. Tregs were cultured either alone (*upper panel*) or co-cultured with CD4+ T effector cells (*middle panel*). The survival of CD4+ T effector cells cultured alone is presented for comparison (*bottom panel*). Results are presented as medians (min.–max.) of the percentages of 7-AAD+ dead cells. Significant differences in Kruskal–Wallis ANOVA between cultures are shown: (*) Tregs without IL2 vs. Tregs with added IL2; (#) Tregs without IL2 vs. cocultures of Tregs and Teffs without IL2; ($) cocultures of Tregs and Teffs without IL2 vs. cocultures of Tregs and Teffs with IL2; (&) Tregs without IL2 vs. Teffs without IL2. **b** In vivo serum concentrations of IL2 were measured during the follow-up. The values for untreated controls (NO Tregs), patients treated with a single infusion (single dose) and patients treated with two infusions (two doses) of Tregs are presented as medians (min.–max.); the *open circles* represent values from particular patients. The p values and *short line* represents significance of Wilcoxon test comparisons immediately after the 2nd Tregs infusion

In the first set of in vitro experiments, a sample of Tregs from the preparation used in the treatment was further cultured with different concentrations of IL2, and cell survival was measured (Fig. 7a). While no supplementation of IL2 was inevitably associated with a rapid decrease in cell survival, relatively low concentrations of IL2, starting from 10 UI/mL were enough to limit cell death (significant from the 2nd day of the culture, KW p < 0.05). IL2 should be present in the culture constantly, since a delayed application of the cytokine had only a minor effect on cell survival. In addition, simple co-culture of Tregs with Teffs without exogenous IL2 significantly improved the viability (significant from 2nd day of the culture, KW p < 0.05). It was additionally synergized when exogenous IL2 was added to the co-cultures. Again, the effect was seen in co-cultures treated with different concentrations of IL2, from the lowest (10 UI/mL) up to the highest (KW p > 0.05 only at the 5th and 7th day).

Dependency of Tregs on IL2 found in vitro was further confirmed by the levels of this cytokine in the sera of patients. Shortly after Tregs infusions, notably in the group treated with two doses of Tregs, concentrations of IL2 transiently decreased, possibly due to the cytokine being metabolized by the infused Tregs (Wilcoxon test: first dose p > 0.05, second dose p < 0.05) (Fig. 7b).

## Discussion

Cellular therapy with autologous expanded Tregs seemed to delay the progression of T1DM but its efficacy could be limited by several factors. As the treatment was introduced after the initial onset of T1DM, probably only a small fraction of β-cells was still preserved in the body and could be protected from autoimmune attack, which was a major limitation of the success of the treatment. The advanced stage of the disease also affected the Tregs. The analysis of this population suggested that it was substantially evolving with the progression of the disease, and it was, therefore, beneficial to use autologous Tregs as early as possible in order to obtain an optimal preparation of Tregs for clinical applications. An additional dose of Tregs later in the study improved the results, but the effects were limited. The disease not only modified Tregs, but also the cytokine milieu towards stronger proinflammatory activity, by increasing the levels of IL6 and, to a lesser extent, TNFα and, IL1. Finally, as confirmed in vitro, survival of the expanded Tregs was dependent on IL2 and the interaction with other lymphocytes.

The time of recruitment to the treatment was an obvious medical limitation of the trial. Current diagnostics identify T1DM patients relatively late, when only 10–30% of the islets are still functional, so only this small fraction can be spared from the disease [11]. Routine markers of autoimmunity in T1DM, such as autoantibodies, may identify patients in danger of disease development. However, their accuracy in prediction of the onset—notably when titers are low or some autoantibodies are not detectable yet—is not sufficient to identify early stage T1DM [12]. It is therefore imperative to develop credible criteria of imminent T1DM, ideally before clinical manifestation, while there is still a substantial mass of β-cells. An enrichment of the routine algorithm of T1DM diagnosis with HLA haplotyping, non-HLA polymorphisms, and an assessment of islet-autoreactive T-cells or circulating DNA of the islets may allow the identification of patients in a very early stage of the disease [13–15]. The administration of Tregs to such patients would definitely improve the success of the therapy.

The need for early intervention is also justified by changes in the Treg population during T1DM progression. We have found that two phenotypes of Tregs, that is, CD3^+^CD4^+^FoxP3^+^ and CD3^+^CD4^+^CD25^high^CD127^−^, overlap, but diverge with time. It implies problems with acquiring Tregs for clinical expansion in the more advanced stage of the disease. It can be performed with either worse purity of putative Tregs or with pure Tregs at the expense of the number of sorted cells. In both cases, this can affect the quality of the preparation that is administered to the patient. Additional proof of the change in the population of Tregs in T1DM was a shifting proportion of naïve and memory Tregs as the disease progressed, notably in the untreated controls. In these subjects, there was a continuous shrinkage of the Tn compartment at the expense of more differentiated memory subsets. Our previous studies suggested that inflammation associated with long-lasting T1DM was responsible for such an effect on Tregs. These cells were characterized by decreased expression of FoxP3 and CD62L under proinflammatory conditions, and this effect could be reverted using anti-inflammatory agents [16–19]. It was intriguing that, in the current study, gradual increase in the level of proinflammatory cytokines was seen early, starting from the clinical onset of T1DM. In addition, it was the only examined immune phenomenon that could not be stopped completely by the adoptive transfer of Tregs.

Interestingly, a swap between naïve and memory Tregs could be also seen in interventional groups immediately after infusion of expanded Tregs. However, it would always revert to the baseline: 2 years after Tregs infusion naïve and memory proportions were close to those observed at the beginning of the study. The patients administered with two doses of Tregs preserved proportions closest to the baseline ratio. This temporary change was probably mostly caused by the content of the administered expanded Tregs, which are usually Tcm FoxP3^high^ Tregs [9]. The fact that they reverted to the baseline ratio in treated subjects suggests a possibility that the level and proportions of Tregs, like other lymphocytes, are homeostatically regulated to some ‘baseline’ levels. Indeed, in our study, the Tcm/Tn proportion of Tregs reverted along with the numbers of total Tregs. Interestingly, the increase in the percentage of Tregs after infusions did not clearly correlate with the dose of administered Tregs, but with C-peptide levels, as shown in our previous report [8]. In addition, the increased level of Tregs, followed by higher C-peptide levels, could be seen after each infusion of the cells. Hence, the number of doses might be an independent factor affecting the efficacy of the treatment, equally important as the cumulative number of Tregs administered. If repetitive doses of Tregs can maintain an increased level of Tregs for a longer period, they can also maintain the C-peptide levels, which was observed in this study in patients treated with two doses of Tregs. The hypothesis on homeostatic regulation is therefore important for the treatment regimen, as splitting the therapy to repetitive smaller doses of Tregs could keep an increased level of Tregs in peripheral blood longer than a single high dose. The assumption on the Tregs homeostat should be completed with the effect of proinflammatory cytokines in T1DM. This proinflammatory activity probably exerts significant influence, as seen in untreated controls, whose sera maintained high levels of proinflammatory cytokines, as well as their Tregs populations were shifted towards the memory subsets in a sustained way.

Finally, we assessed the effect of IL2 on expanded Tregs. This is the cytokine known to influence the survival and function of Tregs [20] and it was also used in high concentrations to expand Tregs for clinical application in this study. The production of the clinical preparation of Tregs required high doses of IL2 and its sudden deprivation on release of the product resulted in a fast decline in cell survival. Fortunately, there were two factors that probably protected Tregs viability in vivo. Even small doses of IL2 added to cell culture—equal to the concentrations of IL2 measured in sera of the patients—significantly improved Tregs viability. Furthermore, the addition of other lymphocytes, which probably secreted IL2 or supported Tregs through cell-to-cell interactions, improved Tregs viability. Most importantly, we found a synergic effect of IL2 and co-cultured Teffs on Tregs viability, since the survival of Tregs in co-cultures with Teffs and exogenous IL2 was comparable to the survival of other lymphocytes. In vivo, the concentration of IL2 decreased shortly after the infusion of Tregs in the group treated with two doses of Tregs, which might simply reflect an increased consumption of IL2 by the infused Tregs. The dependence of Tregs on IL2 was tested in several clinical trials, in which IL2 was either given alone or administered together with expanded Tregs. The former has already been tested in T1DM, but appeared to be ineffective [21]. The latter was tested in graft-versus-host disease with some effect; however, although IL2 as a medicinal product is indicated against tumors, the co-administration of IL2 and Tregs was associated with manifestation of malignancy [22]. Paradoxically, this might be the major threat of systemic injections of IL2 with Tregs, since the exogenous cytokine might disturb trafficking of administered Tregs attracted to local inflammation, as in *insulitis*. It is an important argument as we confirmed that such an accumulation of Tregs in human inflamed tissues exists [23]. Instead of chemotaxis towards inflamed tissues, Tregs injected in combination with exogenous IL2 may impose generalized systemic immunosuppression, facilitating progression of tumors. Hence, such combined interventions should be performed with caution.

## Conclusions

Currently, there are two centers that completed their first studies with Tregs in T1DM [7, 8, 24]. Safety was confirmed in both studies; however, the results are from small cohorts and, therefore, should be interpreted with caution. Still, the results can help design new, improved treatment protocols. As our trial included matched untreated controls, some efficacy could be also confirmed when comparing the data of the treated and untreated subjects. More importantly, we have found factors that most probably affect the efficacy of this therapy. Mainly, it is the advanced stage of the disease, which has a negative impact on the changes in the Tregs compartment. Based on our previous work, as well as this study, we assume that the inflammatory milieu characteristic for the progression of diabetes is responsible for these changes. High dependency of Tregs on IL2 may also influence the results, but this seems to be controlled by the IL2 produced in vivo. Early administration and repetitive doses of Tregs seemed to improve the results. Hence, new trials should take into account these observations in order to improve the efficacy of this approach. These factors were considered in the design of our new currently ongoing trial TregVac2.0 registered as EudraCT: 2014-004319-35 [5], in which two doses of Tregs are administered in a 3-month interval, and patients are recruited in earlier phases of the disease.

## Additional files

**Additional file 1.** Clinical trial protocol.

**Additional file 2.** Statistics.

## Abbreviations

Anti-GAD65: glutamic acid decarboxylase autoantibody
Anti-ZnT8: zinc transporter 8 autoantibody
7-AAD: aminoactinomycin D
BAFF: B cell activating factor
b.w.: body weight
FACS: fluorescence activated cell sorter
GMP: good manufacturing practice
IFNγ: interferon γ
IL: interleukin
IAA: insulin autoantibody
IA2: islet antigen 2 antibody
KW: Kruskal–Wallis ANOVA
MMTT: mixed meal tolerance test
MW: U-Mann–Whitney test
T1DM: type 1 diabetes
Tcm: T central memory
Teff: T effector/conventional cells
Tem: T effector memory
Tn: T naïve
Tregs: T regulatory cells
TNFα: tumour necrosis factor α
VEGF: vascular endothelial growth factor
vs.: versus
