# Supplementary material for: Factors affecting long-term efficacy of T regulatory cell-based therapy in type 1 diabetes
Source: J Transl Med. 2016 Dec 1;14:332. doi: 10.1186/s12967-016-1090-7 (PMC5131539; doi:10.1186/s12967-016-1090-7)
Supplement: Supplementary file 1 — Additional file 1. Clinical trial protocol. [file 12967_2016_1090_MOESM1_ESM.doc]

# STUDY PROTOCOL

# „Cellular Therapy of Type 1 Diabetes with ex vivo expanded CD4+CD25+CD127- T regulatory cells”

Natalia Marek-Trzonkowska VMD PhD

Małgorzata Myśliwiec MD PhD

Piotr Trzonkowski MD PhD

# **Protocol NKEBN/8/2010 (last amendment 01.05.2012):**

**Acronym Title:** TregVac

**Clinical Phase:** Phase 1 [classified by the Ethics Committee] **Accrual Objective: 20 [**10 subjects in Tregs arm and 10 subjects in control arm]

**Accrual Period: 12** months **Follow-up Period: 24** months

**Study duration: 36** months

**Place:** Medical University of Gdańsk, Department of Immunology and Department of Pediatrics, Hematology and Oncology; National Blood Bank, Gdańsk Unit

**Study Design:** A prospective, non-randomised pilot study to investigate the safety and efficacy of cellular therapy of recent-onset type 1 diabetes (DM1) with ex vivo expanded CD4+CD25+CD127- T regulatory cells.

**Treatment Description:** Patients will receive a single or double infusions of ex vivo expanded autologous CD4+CD25+CD127- T regulatory cells (Tregs) in a total dose of up to 30x10^6^cells/kg b.w. All the patients will be on standard insulin therapy. (amended 01.05.2012)

**Primary Endpoint:** Success of the treatment will be defined as a proportion of patients with higher C-peptide and lower requirement for exogenous insulin in the treated group in comparison with the control individuals. The key endpoint is:

- The proportion of subjects with no reported adverse effects of the treatment from day 0 to two years after administration of Tregs. The day of Tregs infusion is designated Day 0 (as recommended by the Ethics Committee).
- The proportion of subjects with daily insulin dose (DDI) ≤ 0.5UI/kg b.w. and plasma fasting C-peptide levels more than 0.5ng/mL present without any stimulation at Day 365. The day of Tregs infusion is designated Day 0.

**Secondary Endpoints:** The key secondary endpoints are the following:

1. The proportion of subjects with daily insulin dose (DDI) ≤ 0.5UI/kg b.w. and plasma fasting C-peptide levels >0.5ng/mL present without any stimulation at 120days after the administration of Tregs. The day of Tregs infusion is designated Day 0.
2. The proportion of subjects with daily insulin dose (DDI) ≤ 0.5UI/kg b.w. and plasma fasting C-peptide levels >0.5ng/mL present without any stimulation from Day 0 to two years after the administration of Tregs. The day of Tregs infusion is designated Day 0.
3. The proportion of subjects with ≥15% reduction of DDI/kg b.w. from baseline at 120days after the administration of Tregs. The day of Tregs infusion is designated Day 0.
4. The proportion of subjects with ≥15% reduction of DDI/kg b.w. from baseline at 2 years after the administration of Tregs. The day of Tregs infusion is designated Day 0.
5. The proportion of subjects with ≥2% reduction of HbA1c from baseline at 120days after the administration of Tregs. The day of Tregs infusion is designated Day 0.
6. The proportion of subjects with HbAlc ≤6.5% at 120days after the administration of Tregs. The day of Tregs infusion is designated Day 0.
7. The proportion of subjects with HbAlc ≤7.0% at 120days after the administration of Tregs. The day of Tregs infusion is designated Day 0.
8. The proportion of subjects with HbAlc ≤6.5% at Day 365. The day of Tregs infusion is designated Day 0.
9. The proportion of subjects with HbAlc ≤7.0% at Day 365. The day of Tregs infusion is designated Day 0.
10. The proportion of subjects with HbAlc ≤6.5% from Day 0 to two years after the administration of Tregs. The day of Tregs infusion is designated Day 0.
11. The proportion of subjects with HbAlc ≤7.0% from Day 0 to two years after the administration of Tregs. The day of Tregs infusion is designated Day 0.

Efficacy Endpoints:

At 120 ± 14 days following the adoptive transfer of Tregs:

- - - - - The proportion of subjects with DDI ≤ 0.5UI/kg b.w.
        - The proportion of insulin-independent subjects
        - C-peptide levels
        - HbAlc levels
        - Quality of life (QOL) measures
        - Functional tests results [can only be performed when indicated by medical conditions of the patients and NOT solely as an efficacy check as recommended by the Ethics Committee]

At 365 ± 14 days following the adoptive transfer of Tregs:

- - - - - The proportion of subjects with DDI ≤ 0.5UI/kg b.w.
        - The proportion of insulin-independent subjects
        - C-peptide levels
        - HbAlc levels
        - Quality of life (QOL) measures
        - Functional tests results [can only be performed when indicated by medical conditions of the patients and NOT solely as an efficacy check as recommended by the Ethics Committee]

At 2 years ± 14 days following the adoptive transfer of Tregs:

- - - - - The proportion of subjects with DDI ≤ 0.5UI/kg b.w.
        - The proportion of insulin-independent subjects
        - C-peptide levels
        - HbAlc levels
        - Quality of life (QOL) measures
        - Functional tests results [can only be performed when indicated by medical conditions of the patients and NOT solely as an efficacy check as recommended by the Ethics Committee]

Safety End Points:

Day of the Treg infusion:

- Assessment of the incidence and severity of immediate Adverse Effects resulted from the administration of Tregs (reactions of the hypersensitivity, embolism) and blood drawing (>2 g/dL decrease in haemoglobin concentration)

14 days following the adoptive transfer of Tregs:

- Assessment of the incidence and severity of possible Adverse Effects resulted from the infusion of Tregs including: viral, bacterial, or fungal infections, and benign or malignant neoplasms,

120 ± 14 days following the adoptive transfer of Tregs:

- Assessment of the incidence and severity of possible Adverse Effects related to the infusion of Tregs including: viral, bacterial, or fungal infections; and benign or malignant neoplasms.

365 ± 14 days following the adoptive transfer of Tregs:

- The incidence and severity of possible Adverse Effects related to the infusion of Tregs including: viral, bacterial, or fungal infections; and benign or malignant neoplasms,

2 years ± 14 days following adoptive transfer of Tregs:

- The incidence and severity of possible Adverse Effects related to the infusion of Tregs including: viral, bacterial, or fungal infections; and benign or malignant neoplasms,

Cessation of the Trial Criteria:

- Any severe Adverse Effect in two or more patients with confirmed link to the administration of Tregs

Inclusion Criteria:

Patients who meet all of the following criteria are eligible for participation in the study:

TREGS STUDY ARM

1. Male and female patients 5 to 18 years of age.
2. Ability to provide written informed consent by parents (and patients if above 16years old)
3. Clinical history of autoimmune type 1 diabetes diagnosed within recent 2 months and presence of at least one type of anti-islet autoantibody: antiGAD, antiIA2, IAA, ICA (high titer),.
4. Fasting plasma C-peptide more than 0.4ng/mL
5. Involvement of the patients and parents in the intensive diabetes management defined as self monitoring of glucose values no less than three times/ day and by the administration of insulin injections each day or insulin pump therapy.
6. Patient and parents mentally stable and able to comply with the procedures of the study protocol.
7. Appropriate venous access for blood drawing.

CONTROL GROUP ARM :

1. Patients fulfilling the inclusion criteria from 1 to 6 but
2. EXCLUDED from blood drawing due to inappropriate venous access.

Exclusion Criteria:

Patients who meet any of these criteria are not eligible for participation in the study:

1. No agreement for participation in the study and no inform consent singed
2. Other than autoimmune type 1 diabetes
3. Age below 5 and above 18 years at the time of recruitment
4. Carriage of HLA-DQB1*0602 allele
5. IgA deficiency or other genetic defect present
6. Body mass index (BMI) outside the range of 25-75 percentiles for a particular age.
7. Presence or history of active infection including hepatitis B, hepatitis C, HIV, syphilis or tuberculosis (TB). Subjects with laboratory evidence of active infection are excluded even in the absence of clinical evidence of active infection.
8. Invasive aspergillus, histoplasmosis, or coccidioidomycosis infection within one year prior to study enrollment.
9. Any history of malignancy
10. Baseline Hb below the lower limits of the reference range ; lymphopenia (<l,000/uL), neutropenia (<l,500/uL), or thrombocytopenia (platelets <100,000/uL).
11. Known hypercoagulative state.
12. Medical treatment requiring chronic use of drugs other than insulin
13. Treatment with any anti-diabetic medication other than insulin within 4 weeks of enrolment (special attention to exclude anti-CD3 treated patients).
14. Diabetic retinopathy.
15. Arterial hypertension.
16. Presence or history of macroalbuminuria (>300 mg/g creatinine).
17. For female subjects older than 15 years positive pregnancy test, unwillingness to use effective contraceptive measures for the duration of the study and 4 months after discontinuation, when appropriate. For male subjects: intent to procreate during the duration of the study or within 4 months after discontinuation or unwillingness to use effective measures of contraception when appropriate.
18. Excessive anxiety of the patient or parents related to the procedures.
19. Any medical condition that, in the opinion of the investigator, will interfere with safe participation in the trial.
20. For parents and children older than 15 years: known active alcohol or substance abuse.

**A. State of the art and objectives:**

**The objectives of the proposal: The aim of this study is to develop efficient cellular vaccine protecting from the diabetes type 1**

**Diabetes – basic epidemiology facts**

Morbidity of diabetes type 1 is getting higher each year in the majority of the developed countries. According to the International Diabetes Federation there were 193 mln of patients in 2003 and this number had increased to 246mln in 2007. Estimated number of diabetic patients in 2030 will be 366mln – around 5% of the human population all around the world will suffer from diabetes. Immune-mediated diabetes type 1 (DM1) consists of around 10-15% of all cases and the increase in this type of the disease is especially dramatic as DM1 affects mainly children and young adults. DM1 is diagnosed in 15/100 000 children in the US. In Finland, where the morbidity is the highest in the world, it is as much as 35/100 000. But DM1 is not only a Finland’s problem as the whole Eastern Europe note systematic increase in the ratio of new onset DM1. With all the development implemented in our side of Europe after communism fall, we have implemented also a burden of all risk factors of autoimmune diseases characteristic for the developed countries of the West. DM1 is a rising problem in Eastern Europe. For example, it is estimated that the morbidity in Poland is around 7/100 000 but in the most affected areas (Pomorskie and Lodz Regions) it is as high as 9.2/100 000, which means that the number of new onset DM1 doubled within 10 years only! (Bodalski J 2000)

**Diabetes as autoimmune disease**

It has been known from 80s’ of the XX century that DM1is an autoimmune disease. First, it has been found that the onset of the disease correlates with so-called insulitis, which is an inflammation in the pancreatic tissue with the presence of activated, self-reactive lymphocytes that infiltrate the pancreatic islets and destroy the insulin-producing beta cells (Gepts W 1981; Eisenbarth GS 1986). Then, it has been confirmed in animal models that the transfer of autoimmune anti-islet T cells from diabetic animals induced insulitis and diabetes in previously healthy animals (Bendelac A 1987). Although there is a long-lasting common agreement that such diabetogenic autoimmune T cells exist also in humans, their presence have been confirmed with tetramer technology only recently (Velthuis JH 2010).

Renewed interest on immunity in DM1 started with the discovery of highly suppressive T regulatory cells (Sakaguchi S 1995) which important role in the pathogenesis of this disease has been confirmed (Salomon B 2000). It has been found in several animal models that CD4+CD25+ T regulatory cells (Tregs) can stop destruction of pancreatic islets and protect from the development of autoimmune DM1 (You S; Green EA 2003). This finding was more difficult to confirm in humans, where the results were ambiguous. Some studies have revealed low level of Tregs in the peripheral blood of DM1 patients (Yang Z 2007), while others did not (Zavattari P 2004; Putnam AL 2005). A proof of principle came from the discovery of IPEX syndrome – recently described inherited disorder related to *Foxp3* gene in which patients do not have functional Tregs (Bennett CL 2001). Immanent features of IPEX (the immune dysregulation, polyendocrinopathy, enteropathy, X-linked) are dysregulation, related to the lack of Tregs, and diabetes, as a one of the endocrinopathies found in the syndrome due to Tregs deficiency. One may assume that similar dysregulation occurs in DM1. In this case, it does not only derives from a number deficiency of Tregs but it is also a result of functional impairment of this subset in DM1 patients (Brusko TM 2005; Lindley S 2005). Indeed, we and others found that in diabetes the proportion of fully functional CD62L+Tregs is decreased at the expense of so-called effector CD62L- Tregs with limited suppressive activity (Ryba M 2009). Hence, it is important to closely follow the phenotype of Tregs in DM1.

**Current immune-based therapies in diabetes**

The knowledge on autoimmune background of DM1 has been used in attempts to diagnose the disease and also to cure it. After few decades of discoveries we can precisely diagnose the patients assessing autoantibodies in their blood, like anti-GAD65, anti-insulin, anti-islets, anti-insulinoma-associated tyrosine phosphatase (IA-2), anti-fogrine and, anti-Zn transporter antibodies. This way we can even catch those in a prediabetic phase, before the clinical onset. Moreover, we can even select those at risk of the development of DM1 using HLA-typing (Rich R.R. 2008).

Such a sophisticated diagnostics can move the treatment of DM1 beyond an ordinary replacement of insulin which is a routine currently. The ultimate goal of the diagnostic process is the selection of a group of patients in which the pancreatic islets are still alive in a relatively high mass. Such patients can then receive treatment to save remaining islets and stop the clinical manifestation of DM1. Currently, there are two main streams of such treatment: with the administration of anti-CD3 antibodies and the manoeuvres encouraging immune tolerance to islets.

The idea of anti-CD3 administration was strictly related to autoreactive anti-islet T cells which destroy insulin-producing islets (Chatenoud L 1989). The antibodies were supposed to kill/deplete or inhibit unwanted effector T cells. In addition, it was found that the therapy enhanced beneficial regulatory T cell functions, thus promoting immune tolerance. Our centre takes a part in phase III trial with this drug. [after protocol: unfortunately, disappointing results of phase III trials and majority of the trials with different anti-CD3 antibodies were suspended]

Induction of immune tolerance is another area of immune-based therapies in DM1. Antigen-specific therapies with intranasal or oral administration of insulin or other peptides supposed to induce tolerance to islets still consists a big proportion of these interventions. In the majority of pre- and clinical studies, in which this strategy proved successful, the tolerance was dependent on Tregs (Staeva-Vieira T 2007). Hence, there are suggestions that simply cellular therapy with Tregs should be effective to stop DM1 (Brusko TM 2008).

**Idea of the current study: T regulatory cells vaccine for diabetes**

Recent development in biotechnology allowed for separation and expansion of Tregs. In our laboratory these cells are examined from the end of XX century and we were able to test all available methods of their separation and expansion (Trzonkowski P 2009b). The attempts to settle this research in the clinical context started in our laboratory in 2004 (Trzonkowski P 2004). In 2009 we achieved first clinical success with in vivo application of these cells [trial registered as NKEBN 458/2007- last amendment]. We achieved a significant reduction in drug doses administered and relieve of clinical symptoms in a group patients with chronic graft versus host disease with the longest remission lasting over two years (Trzonkowski P 2009a). The number of patients recruited to this trial is now eight patients as we are allowed to treat with expanded Tregs only those patients who do not respond to other therapies. Nevertheless, even with so small group in the follow up, we can state that all the patients tolerate well the administration of Tregs and nobody experienced side effects of the therapy.

Alive lymphocytes can be separated either with immunomagnetic kits or using FACS sorters. For several reasons the latter technique seems to be more useful in the work with Tregs in the human setting. Only a small proportion of CD4+ T cells with the highest expression of CD25 receptor and without CD127 receptor appears to have regulatory properties in humans. Purity of the preparation can be additionally improved with some other manoeuvres possible only with FACS sorters, such as doublet exclusion and multicolour phenotyping. With more than twenty different receptors checked in our laboratory, CD127-CD25highCD4+CD3+Lin-doublet- phenotype appeared to be optimal for clinical applications, notably for expansion under GMP conditions (Trzonkowski P 2009b).

When sorted, Tregs need to be expanded in order to be transferred to the patient. First of all, it requires certified GMP laboratory and the expansion procedure itself needs to be certified as well. It means that each and every element of the procedure including equipment and consumables needs to be checked and approved. At the same time, cultures of Tregs ex vivo are fragile and very easy decrease suppressive abilities. With all the efforts we were able to achieve stable cultures with good suppressive potential and now we are able to prepare preparation of Tregs approved according to the GMP requirements for patients. These requirements were already implemented in our university and the University of Chicago who cooperates with us (Marek N 2010). The quality of Tregs preparation easily decreases, mainly due to the time outside the body. Importantly, we found that both GMP conditions and reduction in the time of expansion are crucial for the success (Marek N 2010). Thorough analysis of the cultures of Tregs during expansion allowed us to optimize the procedure, so the suppressive abilities of these cells can be preserved throughout the whole expansion and patients can receive the product with more than 90% activity measured with FoxP3 expression. Importantly, in the two recently presented trials (ASH 2010) in which patients were treated with expanded Tregs the purity of the final product administered was around 60% and in some cases as low as 20% - close to the percentage of FoxP3 expression in total CD4 T cells [trials are now published: Brunstein CG 2011; Di Ianni M 2011].

**B. Procedures**

**All procedures are concordant with ‘the Medical Experiment requirements’ chapter of the Medical Doctor Act (Parliament of the Republic of Poland 1999 with amendments)**

1. **Recruitment**

The recruitment will be performed in the outpatient clinic of the the Department of Pediatrics, Hematology and Oncology Medical University of Gdańsk. Patients and parents will have a chance to discuss the study procedures and options with the recruiting named physician and time to read all necessary information related to the study (attached to this document as information for patients and parents).

1. **Blood drawing**

Recruited patients, after informed consent is signed by the parents, will be admitted to the Department of Pediatrics, Hematology and Oncology Medical University of Gdańsk. Blood will be drawn in the clinic by certified nurse according to the standard operating procedure of the ward for handling of blood samples. The only exception is the need of anastesiologist assistance during the procedure.

The volume of blood drawn will be calculated according to the following algorithm: Maximal volume of blood to be drawn is equal to 1% of the body weight but no more than 250ml [e.g. child weighting 20kg – 200ml of blood is allowed, child weighting 40kg – 250ml of blood is allowed ]. Then it will be separated in the blood bank to buffy coat and plasma and transferred to GMP laboratory [details of the transfer described in standard operating procedures for blood transport of the blood bank] for further processing.

**3. Production of Tregs**

T regulatory cell will be produced from autologous peripheral blood taken from patients. The procedure of the GMP laboratory are described in the Standard Operating Procedures of GMP laboratory and certification forms.


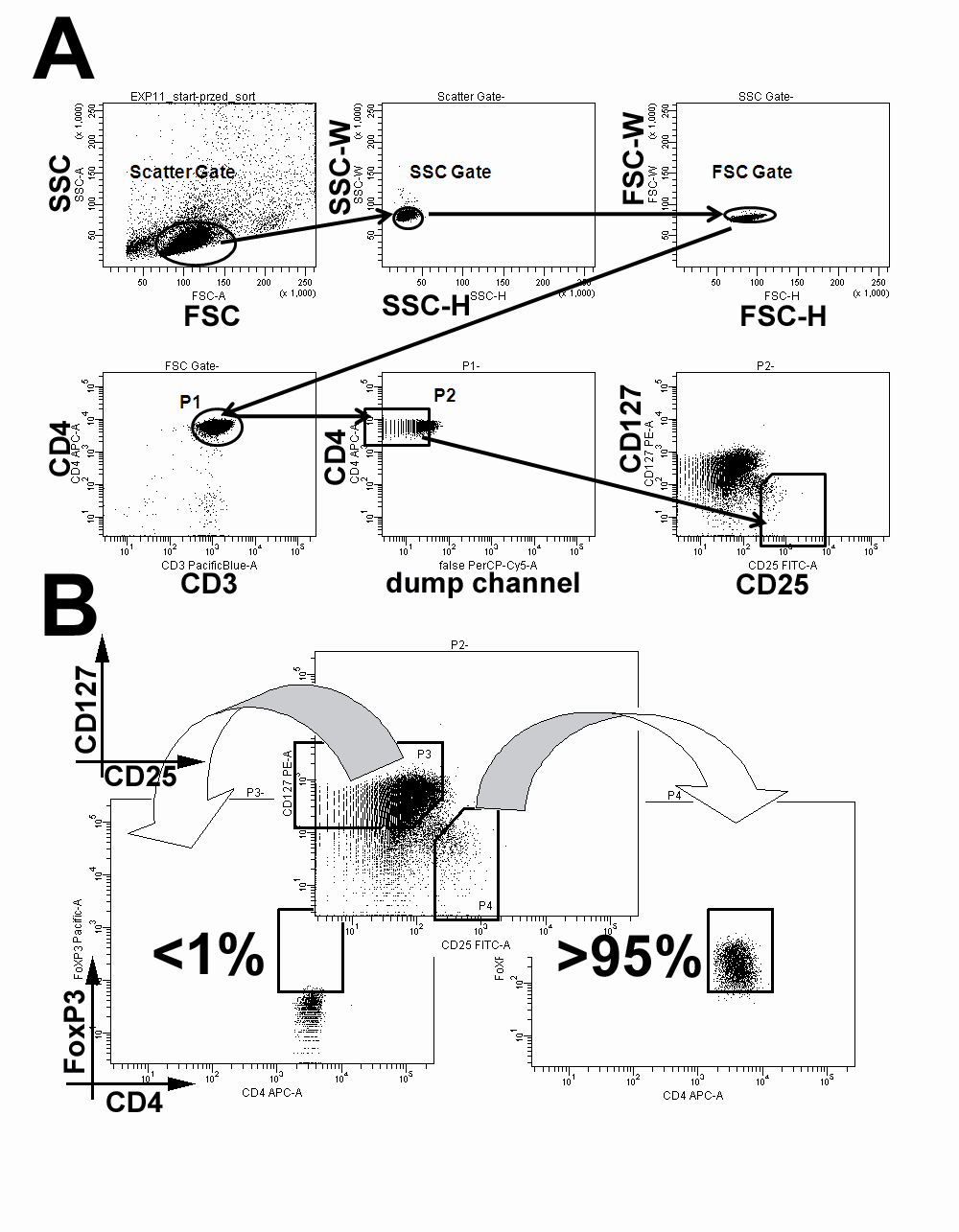
**3.2. Tregs sorting and expansion**

Tregs and their subsets will be separated using compilation of FACS sorting and immunomagnetic techniques. Then, the cells will be cultured and expanded in sterile conditions of our GMP clean laboratory certified for the preparation of human tissues.

**3.2.1. Tregs sorting**

Figure B.3.1: Gating strategy for the sorting of Tregs. Cells will be presorted with GMP-approved CD4+ immunomagnetic kit and then sorted by FACS. Cells will be sorted with Influx cell sorter which is equipped in single-use sample and fluidics lines, HEPA enclosure and UV sterilizing. As that, this makes it possible to approve the machine as GMP-grade equipment.

**A.** Lymphocytes in FACS sorter will be identified using a routine scatter gate procedure. First, the doublets (cell conglomerates stuck to specifically stained cells and responsible for poor sort purity) will be eliminated. This will be accomplished using laser light signal characteristics (width and height) with two subsequent gates: the side scatter gate and the forward scatter gate (SSC-W vs. SSC-H and FSC-W vs. FSC-H dot plots, respectively). Events from the later gate will be then transposed to the CD3 vs. CD4 dot plot (P1) to obtain CD4+ T cells only. Before the final gating, potential dead cells and non-CD4 remnants from the P1 gate will be excluded using a dump channel (P2 gate). In addition to specific staining, the cells will be stained with CD8, CD19, CD16, and CD14 PerCP-conjugated antibodies. Because all of the fluorescence from the cells stained with dump channel antibodies will be detected by the same PMT sensor, it will be possible to gate out unwanted events in a single step. Finally, the cells from P2 gate will be transposed to the CD127 vs. CD25 dot plot and the top 2% of the CD25+ T cells, which are CD127-, will be sorted.

**B.** Purity is an absolute priority in the procedure and the presented gating procedure gives almost 100% efficiency, as we have confirmed in our lab. The upper dot plot is generated from human CD4+CD3+ T cells during the sorting procedure. Anti-CD127 staining during phenotyping shows that the CD4+ T cells with the highest CD25 receptor expression are devoid of CD127 expression. Moreover, reanalysis of sorted subsets, shown in the bottom dot plots, shows that more than 95% of the sorted CD127negativeCD25highCD4+CD3+ T cells (P4 gate, right bottom dot plot) express FoxP3, while the majority of CD127+CD25low/negativeCD4+CD3+ T cells are negative for FoxP3.

Histograms are from FACS Aria which will be used only for the quality check in the current study.

**3.2.2. Tregs expansion**


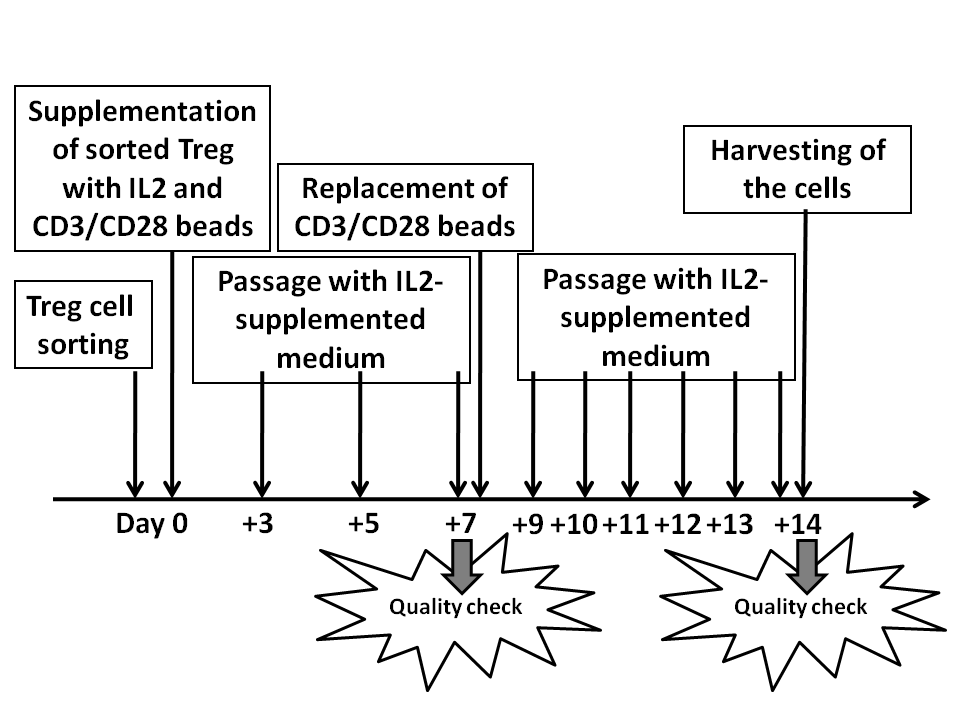
Figure B.3.2: Ex vivo expansion of Tregs

The flowchart outlines the procedure, starting from sorting of the Treg cells.

CD127^low^CD25^high^CD4^+^CD3^+^ sorted Tregs will be cultured in 96-well U-bottom plates. Tregs (1 × 10^5^ per well) will be cultured in a high dose of IL2 (1,000 IU/ml) and anti-CD3/CD28 beads in 1:1 ratio in autologous heat-inactivated serum. Importantly, all media and cytokines will keep GMP-grade certificate. The cells will be passaged every two days during the first week and every day during the second week with new medium supplemented with IL2. On day +7, the beads will be replaced with a fresh set (one bead per cell). The number of cells increases exponentially and the desired number of cells is usually achieved between 7^th^ to 14 day of the culture. At the day +7 and at the end of the culture, the product is checked for quality as described below. The culture is stopped either when the desired number of cells is achieved or at the day +13 of the culture. Cells are washed out from expansion beads and IL2 and suspended in medium with 10% autologous serum for 24 hours. After this time certification is performed and the cells are suspended in 250ml of 0.9% NaCl and closed in thermostable container in order to be released. These conditions are also published in Cell Transplantation journal (Marek N 2010).

**3.3. Certification of the product**

In order to be released, the product must fulfil requirements for cellular therapy according to the Polish Transplantation Act 2007 and Directive 1394/2007 of the European Parliament. Release criteria are:

1. FoxP3 level at least 75% in any time point of 14 days of the culture
2. Confirmed suppression of IFNγ production by autologous effectors
3. Negative results of microbiological tests

**3.3.1. Quality check**

It is important to prove that the preparation is a defined cell subset with suppressive abilities. Therefore, the culture will be thoroughly monitored during the expansion and immediately before administration. Purity of the product will be checked with phenotyping and FoxP3 staining. For the functional assessment we have developed quick IFN-suppression assay, which is 5-hours-lasting alternative to 5-days-lasting proliferation suppression assay. ***This way the result is available at the time of Tregs release from the laboratory*** (Trzonkowski P 2004; Marek N 2010).


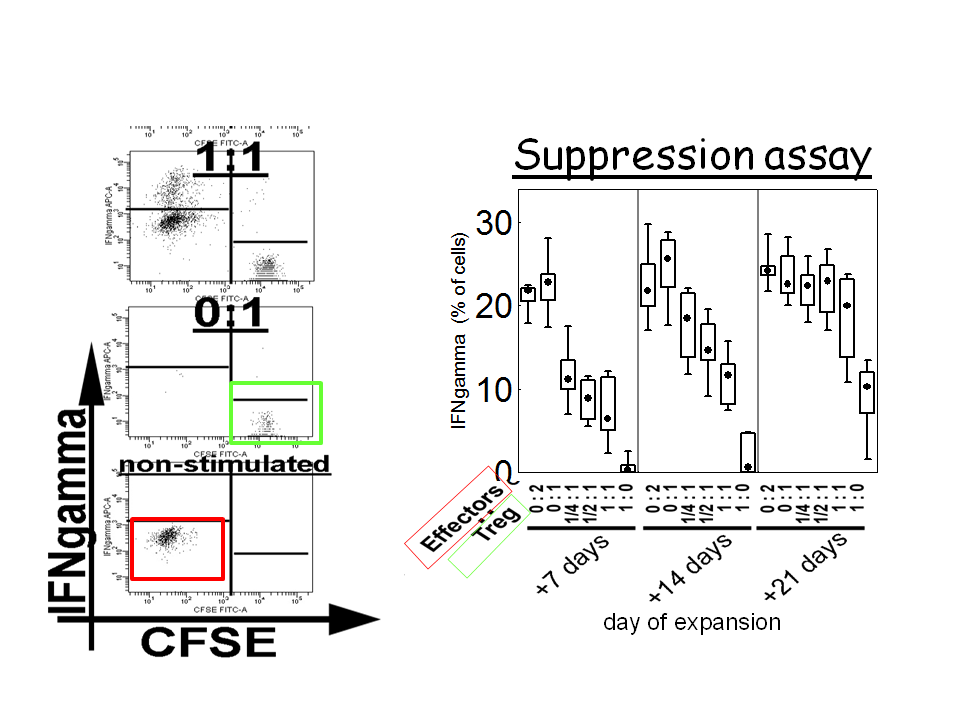
Figure B.3.3: Quick IFN-suppression assay. Samples of Tregs from the cultures will be cocultured with autologous effectors in different concentrations of Effectors : Tregs (‘X’ axis) and then the cocultures will be stimulated to produce IFNγ. Immediately prior to the assay Tregs will be stained with cell tracer CFSE in order to distinguish them from effectors and therefore it was possible to give separately the proportions of IFNγ-positive Tregs (in green rectangle) and effectors (in red rectangle) at the end of the

assay. Examples of the dot-plots in the left column; fluorescence of anti-IFNγ mAb on the ‘Y’ axis with the cut-offs of positive signal based on isotype controls; CFSE signal on the ‘X’ axis with effectors as CFSE-low cells and Tregs as CFSE-high cells; proportions of Effectors : Tregs are given with numbers. Right column gives the example of the assay during different time points of the expansion. The results are presented after subtraction of the values of the background from non-stimulated cultures. The results throughout the figure are presented as medians (symbols inside the boxes), 20-80% percentiles (boundaries of the boxes) and minimum-maximum (error bars outside the boxes).

**3.3.2. Microbiological safety of the procedure**

The cells will be continuously checked during the culture for their biological safety. Cultures for the presence of bacteria, measurements of endotoxin level in the supernatants and the tests for the presence of viral genetic material (HIV, HBV, HCV and CMV+ early CMV antigen) will be performed as a part of the above mentioned “quality check”.

**4. Tregs infusion**

Tregs preparation is released from GMP facility as a cell suspension in 0.9%NaCl and must be administered within one hour from the release. The infusion will be performed in Department of Pediatrics, Hematology and Oncology Medical University of Gdańsk under requirements of the standard transfusion of blood products described in the standard operating procedures of the ward. The preparation of Tregs will be solved in 250ml of 0.9% NaCl and slowly infused with fluidics line with the filter for blood products. At the end of infusion, the line will be switched to the new 100ml container with 0.9% NaCl in order to wash out cells remaining in the line. The procedure can be done by certified nurse with the assistance of the anaesthesiologist and paediatrician named in the protocol or by anaesthesiologist and paediatrician named in the protocol.

**5. Follow up of the patients**

Patients will be examined at recruitment, the day of blood drawing for Tregs expansion, at the day of Tregs infusion and two weeks later, every two months for the first half a year, then every 3 months for the duration of the study (2 years). In each point of the study 15ml of venous peripheral blood will be taken: 8ml on EDTA for cellular analyses (flow), 5ml without anticoagulant for serum and 2 ml for DNA.

Control patients – after recruitment blood taken during routine visits only.

**Follow-up visit schedule**

|  | **R^1^** | **A^2^** | **0d^3^** | **+14d** | **+2m** | **+4m** | **+6m** | **+9m** | **+1y** | **+15m** | **+18m** | **+21m** | **+24m** |
| --- | --- | --- | --- | --- | --- | --- | --- | --- | --- | --- | --- | --- | --- |
| Clinical examination | x | X | x | x | x | x | x | x | x | X | X | x | x |
| Insulin requirement | x | X | x | x | x | x | x | x | x | X | X | x | x |
| HbA1c (%) | x | X | x | x | x | x | x | x | x | X | X | x | x |
| C-peptide | x | X | x | x | x | x | x | x | x | X | X | x | x |
| Quality of life | x |  | x |  | x |  | x |  | x |  | X |  | x |
| Treg phenotype | x |  | X | x | x | x | x | x | x | X | X | x | x |
| Autoantibodies | x |  | X | x | x | x | x | x | x | X | X | x | x |
| Cytokines | x |  | X | x | x | x | x | x | x | X | X | x | x |

1 – recruitment

2 – Blood drawing

3 – Tregs infusion

**5.1. Metabolic Assessments**

**5.1.1. Insulin requirements**

Subjects will record their total daily insulin dose on self-monitoring diaries. Subject should be given exogenous insulin as needed to maintain fasting capillary glucose levels <140 mg/dL (7.8mmol/L) at a minimum of 4 out of 7 days a week; 2-hour post-prandial capillary glucose levels should not exceed 180 mg/dL (10.0 mmol/L) more than 3 times per week.

**5.1.2. Glycemic control**

Glycemic control will be assessed by HbAlc (%).

**5.1.3. The C-peptide and C-peptide/ (glucose x creatinine) ratio**

The C-peptide levels will be assessed routinely. Functional tests can only be performed if required in the routine treatment of the patient.

Alternatively, C-peptide/ (glucose x creatinine) ratio (CPGCR) might be considered from the fasting (basal) glucose and c-peptide, and a simultaneous serum creatinine. This measure accounts for both the dependence of c-peptide secretion on the ambient glucose concentration and the dependence of c-peptide clearance on kidney function. The CPGCR is calculated as [c-peptide (ng/mL) * 100]/[glucose (mg/dL) * creatinine (mg/dL)]. An index of islet function, this measure correlates well with both the 90-minute glucose levels during a MMTT and the Beta-score.

**5.1.4 Quality of life**

Generic and disease-specific measures adapted from other studies will be used to assess quality of life.

Generic Measures

Version 2 of the SF-36® Health Survey, standard (4-week) recall form.

This widely used, generic instrument derives eight scales (physical functioning, role-physical, bodily pain, general health, vitality, social functioning, role-emotional, mental health) and two summary components (physical and mental). If the 36-item version of the instrument were felt to be too lengthy, version 2 of the SF-12 (standard recall form) would be an option. This shorter version would derive eight scales and two summary components and would be also be normed to the 1998 data (general population and disease-specific groups).

Disease-targeted Measures

Diabetes Distress Scale

The Diabetes Distress Scale (DDS) represents the latest iteration of the Problem Areas in Diabetes (PAID) scale. This is a 17-item self-administered questionnaire culled from a longer battery of 28 items. Psychometric properties for the DDS were previously published in Diabetes Care. The DDS measures four diabetes-related distress domains: emotional-burden (EB), physician-related interpersonal distress (PD), regimen-related distress (RD), and diabetes-related interpersonal distress (ID). Internal consistency as measured by Cronbach's coefficient alpha ranged between 0.88 and 0.93 for the multi-item scales. The developers tested for and demonstrated construct validity using exploratory factor analysis.

**5.2. Immunologic Testing**

**5.2.1. HLA typing**

HLA typing will be performed on recruitment. All the HLA tests will be performed using SSP-PCR assays.

• material- patient’s whole blood

**5.2.2. Cellular immunity and regulation**

The pholowing phenotype will be screened for the assessment of T regulatory cells: CD3/CD4/CD25/CD127/CD45RA/CD62L/FoxP3

• material- patient’s leukocytes

**5.2.3. Autoantibodies**

The level of autoantibodies is the best known feature of immune response with confirmed link to the autoreactivity to islets. The most common anti-islet antibodies, such as antiGAD, antiIA2, IAA, ICA high titer, antiZnT8, will be followed on protocol for the duration of the study (2 years).

• material- patient’s serum

**5.2.4. Cytokines**

In order to correlate expression of proinflammatory cytokines blood will be collected for assessment of proinflammatory and anti-inflammatory cytokines such as IL1, IL6, IL8, TNFα, IFNs, IL17, IL10, TGFβ.

• material- patient’s serum

**5.3. Archived serum and plasma**

In order to ensure that we will ultimately gain as much information as possible from these trials, and due to the ongoing development of assays such as T cell assays, serum, cells and RNA will be archived for future analyses. Details for subjects regarding the archiving of samples and use for future assays are contained in the study's informed consent form. Subjects will have the option of whether or not they want to have samples archived and will indicate their choice on the informed consent form. A subject's choice regarding archiving samples will not affect his/her participation in the study.

Serum: Blood will be collected to obtain serum and archived.

DNA: Blood will be collected to obtain DNA, processed and archived.

C. Human Resources for the project

|  | Key team members | Role |
| --- | --- | --- |
| 1. | Piotr Trzonkowski,  MD PhD  Professor of Immunology | Project coordinator, GMP facility director (processing), Work with Tregs, sorting, flow cytometry and cell culture techniques expertise |
| 2. | Małgorzata Myśliwiec, MD PhD  Professor of Pediatrics | Head of the Department of Pediatrics, Hematology and Oncology Medical University of Gdańsk, Inclusion and clinical follow-up of the patients |
| 3. | | Piotr Witkowski,  MD PhD,  Assistant Professor of Surgery | | --- | | Director of Pancreatic Islets Transplanation at the University of Chicago, USA; External advisor of the Gdańsk programme, GMP requirements advisor |
| 4. | Wojciech Młynarski, MD PhD  (Professor of Pediatrics) | Inclusion and clinical follow-up of the patients |
| 5. | Natalia Marek-Trzonkowska,  VMD PhD  (Immunology and Diabetes) | Research fellow; Work with Tregs, sorting, flow cytometry and cell culture techniques expertise |
| 6. | Marcelina Grabowska, MSc, (Clinical diagnostics) | Clinical diagnostics, follow up of the patients |
| 7. | Anita Dobyszuk MSc (Biology) | GMP specialist, Clinical Product Certification officer |
| 8. | Ilona Techmańska, MD (Diabetology) | Inclusion and clinical follow-up of the patients |
| 9. | Agnieszka Brandt, MD (Diabetology) | Inclusion and clinical follow-up of the patients |
| 10. | Jolanta Juścińska, MD PhD (Transfusiology) | Blood product specialist |
| 11. | Magdalena A. Wójtewicz, MD  (Anaesthesiology) | Anaesthesiological care |
| 12. | Radosław Owczuk, MD PhD  Professor of Anaesthesiology | Anaesthesiological care |
| 13. | Lucyna Sharma, MSc  ( Chief Nurse) | Inclusion and clinical follow-up of the patients, Blood drawing certificate |
| 14. | Anna Balcerska MD PhD  (Professor of Pediatrics) | Internal advisor of the programme |
| 15. | Jolanta Myśliwska MD PhD  (Professor of Immunology) | Internal advisor of the programme |

**D. Safety Monitoring for phase I study** (as recommended by the Ethics Committee)

1. **Clinical safety assessments**

Clinical safety will be assessed by physical examination, measurement of vital signs (blood pressure, heart rate, oral temperature and respiratory rate) and occurrence of clinical events such as opportunistic infection and other reportable adverse events. These assessments will be made at regularly scheduled clinic visits at intervals detailed in schema of the follow up.

1. **Product contamination**

Physician notification of Tregs product contamination occurs as dictated by Microbiology Laboratory SOP by direct phone notification of the principal investigator (PI) for positive culture results. The PI’s mobile number is included in the SOP for this protocol, available to technicians in the Microbiology Laboratory.

Upon learning of contamination in a batch already administered, the patient will be contacted by telephone by PI, the nurse coordinator, or another co-investigator, informed of the contamination, and queried regarding symptoms including but not limited to fever, chills, malaise and dizziness. Negative responses will result in verbal reassurance, counseling on signs/symptoms which should prompt the subject to contact our center and/or seek medical assistance, and the addition of a complete blood count (including white blood count and differential) and blood cultures x 2 at the next protocol-scheduled blood draw. Affirmative responses to any of these questions will result in requesting the subject to come immediately to The Medical University of Gdańsk Medical Centre, or, if not possible, to another medical facility for evaluation. Evaluation will include at least history and physical, CBC and blood cultures. Fever, leukocytosis, and/or positive blood cultures will result in hospitalization and the initiation of empirically chosen antibiotics (based on the positive culture results from the product) until the signs/symptoms resolve and/or positively identified organisms with antibiotic sensitivities allow specifically tailored antibiotic therapy.

The fluidics line from infusion is stored for 48 hours and will be sent to the Microbiology Laboratory for assessment when contamination or other adverse effects are reported.

The Ethics Committee will be notified in writing within 30 days of the discovery of contamination in infused batch of Tregs. Notification will be within 48 hours if such contamination results in a serious adverse event.

1. **Data safety and monitoring plan**
   1. **Consenting process**

All parents will have the cognitive ability to provide written consent. The information will be also given to the patients and the receive will be assessed and monitored. Assurance that the patient and parents understands the study will be obtained by routine evaluation of subjects’ mental capacity as part of their routine physical examination. Informed consent is completed by physician and nurses on a routine basis by asking general questions to ensure the patient and parents are oriented to person, place and time. Additional questions will be asked and answers evaluated to discern the level of understanding of the patient and family with regard to what has and will transpire during the course of the treatment process. This will occur repeatedly during each follow-up visit during the course of the study. Consent will be discussed during the initial clinic visit and before any study procedure commences, only after the patient has fulfilled eligibility criteria and is considered by the principal investigator to be an appropriate subject for the study.

- 1. **Consent and study procedure changes**

Any and all changes in the consent form and study protocol will be submitted to the the Ethics Committee of the medical University of Gdańsk and implemented only after receiving written acceptance for the proposed ammendments.

- 1. **Monitoring for toxicity and adverse outcomes**

The schedules for regular physician encounters and laboratory testing are detailed previously. In addition to regular review of this data by the principal investigator and clinical research nurse, the study team will meet at intervals to review individual patient data and summary descriptive statistics on adverse events and measurements of treatment efficacy. Minutes will document each of these conferences and can be presented to the Ethics Committee for external evaluation.

- 1. **Confidentiality of data**

Patients’ identity will remain confidential throughout the study period unless disclosure is required by law. It has been included in the Informed Consent Form that the data may be shared by study personnel other than the Primary Investigator, representatives of the approved government agencies, the Ethics Committee, and other regulatory agencies. Patients will be identified only by a unique identifier number. All data records and computers containing information about subjects specifically relevant to this study will be maintained in a locked, secured office. All study results for studies performed by hospital laboratories for which the results are posted on the Hospital computer system will be maintained by a secured password system with all the confidentiality safeguards afforded to patients receiving routine, non-experimental care. The results of this study may be published and presented in scientific meetings, however, the patient will not be identified in such publications or presentations.

- 1. **Adverse effects**

Definition of adverse event: any unfavorable and unintended sign (including an abnormal laboratory finding), symptom, or disease temporally associated with the use of a medical treatment or procedure regardless of whether the sign, symptom or disease is considered related to the medical treatment or procedure.

Definition of serious adverse event (SAE): any event that is fatal or life-threatening, that is permanently disabling, requires or extends hospitalization of the subject, represents a significant overdose or breach of protocol, or suggests that a drug or procedure used in a research protocol has produced congenital anomaly or cancer, or, in the opinion of the investigator, represents other significant hazards or potentially serious harm to the research subject or others.

Severity grading scale:

Mild: adverse event of little clinical significance

Moderate: adverse event of mild to severe significance; causing some limitation of usual activities

Severe: see definition of SAE above

Attribution scale:

Not related: clearly not related to the study

Possible: may be related to the study

Probable: likely related to the study

Definite: clearly related to the study

Unable to assess

**3.5.1. Adverse event reporting plan**

The principal investigator, together with the Ethics Committee, will serve as the data safety monitor for this study. The principal investigator will provide interim reports as required to the Ethics Committee, and to the General Pharmaceutical Inspectorate. All fatal or life-threatening events occurring as part of this trial will be reported within 48 hours, even if all the information is not available. Follow-up written notification will be submitted within 10 days. All other adverse events will be reported within ten days.

**3.6 Statement of Compliance**

This clinical study will be conducted according to ‘the Medical Experiment requirements’ chapter of the Medical Doctor Act (Parliament of the Republic of Poland 1999 with amendments), Polish Transplantation Act (Parliament of the Republic of Poland 2007) and Directive 1394/2007 of the European Parliament.

Any amendments to the protocol or to the consent materials must also be approved by the ethics Committee of The Medical University of Gdańsk before they are implemented.

**E. Information on materials used in the study**

The study will not use any newly synthesised substances. All used materials and equipment must be certified and appropriately checked for safety.

Diagnostic products - the study will be based on routine plastic labware used in the ward for clinical diagnostics (IVD certified)

Drug products - the study will be based on routinely used insulin preparations, anti-shock kits registered as drugs by the Major Pharmaceutical Inspectorate (pharmaceutical product certified)

Treg infusion – the study will use standard blood drawing kits (triple bag kits) registered by the Hematology and Transfusiology Institute for blood drawing (blood handling certified)

Tregs product

| **Component** | Description |
| --- | --- |
| CD3+CD4+CD25+CD127+ FoxP3+ T regulatory cells | Released after quality check, at least 75% purity as checked by FoxP3 expression, amount up to 20x10^6^ per kg b.w. in 0.9% NaCl (pharma grade) in a single dose (AMENDED 01.05.2012) |
| Intermediate components (trace amounts possible) |  |
| Autologous inactivated serum | Separated in sterile conditions at the time of blood drawing and used as medium supplement (10%) after 30min heat inactivation, washed out at the end of the culture by centrifugation |
| SCGM Medium (CellGro) | CellGro medium designed for GMP grade cell cultures, sterile, endotoxin-free, washed out at the end of the culture by centrifugation, |
| CD3/CD28 bead expanders (Dynal) | GMP-grade artificial beads used to stimulate cell proliferation, sterile, endotoxin-free, washed out at least 24 hours prior to the end of the culture by immunomagnetic separation |
| Plastic labware (Falcon) | The cultures will be performed on plastic labware provided by BD Falcon; all equipment is certified as sterile, endotoxin-free |
| Fluidics kit for influx sorter (BDBioscience) | Single-use sterile endotoxin-free fluidics line used for cell sorting |
| Antibodies for sorting phenotype: CD3/CD4/CD25/CD127/CD14/CD16/CD19/CD8 | IVD-grade antibodies from BDBioscience used for sorting |

**References**

Bendelac A, Carnaud C, Boitard C, Bach JF (1987). "Syngeneic transfer of autoimmune diabetes from diabetic NOD mice to healthy neonates. Requirement for both L3T4+ and Lyt-2+ T cells. ." J Exp Med. **166**: 823–832.

Bennett CL, Christie J, Ramsdell F, Brunkow ME, Ferguson PJ, Whitesell L, Kelly TE, Saulsbury FT, Chance PF, Ochs HD (2001). " The immune dysregulation, polyendocrinopathy, enteropathy, X-linked syndrome (IPEX) is caused by mutations of FOXP3." Nat Genet. **27**: 20-21.

Bodalski J (2000). "Opieka nad dziećmi i młodzieżą chorymi na cukrzycę – aktualne problemy." Przewodnik Lekarza **9**: 14-17.

Brunstein CG, Miller JS, Cao Q, et al. (2011). "Infusion of ex vivo expanded T regulatory cells in adults transplanted with umbilical cord blood: safety profile and detection kinetics." Blood **117**(3): 1061-70.

Brusko TM, Putnam AL, Bluestone JA. (2008). "Human regulatory T cells: role in autoimmune disease and therapeutic opportunities." Immunol Rev. **223**: 371-90.

Brusko TM, Wasserfall CH, Clare-Salzler MJ, Schatz DA, Atkinson MA. (2005). "Functional defects and the influence of age on the frequency of CD4+ CD25+ T-cells in type 1 diabetes." Diabetes **54**(4): 1407-14.

Chatenoud L, Ferran C, Bach JF (1989). "In-vivo anti-CD3 treatment of autoimmune patients." Lancet **334**(8655): 164.

David Kägi, Alexandra Ho, Bernhard Odermatt, Arsen Zakarian, Pamela S. Ohashi and Tak W. Mak,. (1999). "TNF receptor 1 ß cell toxicity dependent as an effector pathway in autoimmune diabetes." J. Immunol. **162**(4598-4605).

Di Ianni M, Falzetti F, Carotti A, Terenzi A, Castellino F, Bonifacio E, Del Papa B, Zei T, Ostini RI, Cecchini D, Aloisi T, Perruccio K, Ruggeri L, Balucani C, Pierini A, Sportoletti P, Aristei C, Falini B, Reisner Y, Velardi A, Aversa F, Martelli MF. (2011). "Tregs prevent GVHD and promote immune reconstitution in HLA-haploidentical transplantation." Blood **117**(14): 3921-8.

Ehrenstein MR, Evans JG, Singh A, Moore S, Warnes G, Isenberg DA, Mauri C. (2004). "Compromised function of regulatory T cells in rheumatoid arthritis and reversal by anti-TNFalpha therapy." J Exp Med. **200**(3): 277-85.

Eisenbarth GS (1986). "Type I diabetes mellitus. A chronic autoimmune disease." N Engl J Med **314**(21): 1360-1368.

Gepts W, Lecompte PM (1981). "The pancreatic islets in diabetes. ." Am J Med. **70**: 105–115.

Green EA, Gorelik L, McGregor CM, Tran EH, Flavell RA. (2003). "CD4+CD25+ T regulatory cells control anti-islet CD8+ T cells through TGF-beta-TGF-beta receptor interactions in type 1 diabetes." Proc Natl Acad Sci U S A. **100**(19): 10878-83.

Gwozdziewiczová S, Lichnovská R, Ben Yahia R, Chlup R, Hrebícek J. Biomed Pap Med Fac Univ Palacky Olomouc Czech Repub. 2005;149(1):109-117. (2005). "TNF-alpha in the development of insulin resistance and other disorders in metabolic syndrome." Biomed Pap Med Fac Univ Palacky Olomouc Czech Repub. **149**(1): 109-117.

Gwozdziewiczová S, Lichnovská R, Hrebícek J. (2004). "Tumor necrosis factor alfa (TNFalpha) and insulin resistance." Cesk Fysiol. **53**(4): 167-175.

Hurlimann D, Forster A, Noll G, Enseleit F, et al. (2002). "Anti-tumor necrosis factor-alpha treatment improves endothelial function in patients with rheumatoid arthritis." Circulation **106**(2184-2187).

Klimiuk PA, Sierakowski S, Domyslawska I, Fiedorczyk M, Chwiecko J. (2004). "Reduction of soluble adhesion molecules (sICAM-1, sVCAM-1, and sE-selectin) and vascular endothelial growth factor levels in serum of rheumatoid arthritis patients following multiple intravenous infusions of infliximab." Arch Immunol Ther Exp (Warsz) **52**: 36-42.

Lindley S, Dayan CM, Bishop A, Roep BO, Peakman M, Tree TI. (2005). "Defective suppressor function in CD4(+)CD25(+) T-cells from patients with type 1 diabetes." Diabetes **54**(1): 92-9.

Lugering A, Schmidt M, Lugering N, Pauels HG, Domschke W, Kucharzik T. (2001). "Infliximab induces apoptosis in monocytes from patients with chronic active Crohn's disease by using a caspasedependent pathway." Gastroenterology **121**: 1145-1157.

Marek N, Bieniaszewska M, Krzystyniak A, Juscinska J, Mysliwska J, Witkowski P, Hellmann A, Trzonkowski P. (2010). "The time is crucial for exvivo expansion of T regulatory cells for therapy." Cell Transplant. in press DOI: 10.3727/096368911X566217.

Nadkarni S, Mauri C, Ehrenstein MR. (2007). "Anti-TNF-alpha therapy induces a distinct regulatory T cell population in patients with heumatoid arthritis via TGF-beta." J Exp Med. **204**(1): 33-39.

Putnam AL, Vendrame F, Dotta F, Gottlieb PA. (2005). "CD4+CD25high regulatory T cells in human autoimmune diabetes." J Autoimmun. **24**(1): 55-62.

Rich R.R., Fleisher T.A., Shearer W.T., Schroeder H.W. Jr, Frew A.J., Weyand C.M. (2008). "Clinical Immunology. Principles and Practice." **III wyd.**(Elsevier): 1035.

Ryba M, Hak L, Zorena K, Myśliwiec M, Myśliwska J. (2009). "CD4+Foxp3+ regulatory T lymphocytes expressing CD62L in patients with long-standing diabetes type 1." Centr Eur J Immunol. **34**(2): 90-93.

Sakaguchi S, Sakaguchi N, Asano M, Itoh M, Toda M. (1995). " Immunologic self-tolerance maintained by activated T cells expressing IL-2 receptor alpha-chains (CD25). Breakdown of a single mechanism of self-tolerance causes various autoimmune diseases." J Immunol. **155**(3): 1151-64.

Salomon B, Lenschow DJ, Rhee L, Ashourian N, Singh B, Sharpe A, Bluestone JA. (2000). "B7/CD28 costimulation is essential for the homeostasis of the CD4+CD25+ immunoregulatory T cells that control autoimmune diabetes." Immunity **12**(4): 431-440.

Staeva-Vieira T, Peakman M, von Herrath M. (2007). "Translational mini-review series on type 1 diabetes: Immune-based therapeutic approaches for type 1 diabetes." Clin Exp Immunol. **148**(1): 17-31.

Tak PP, Taylor PC, Breedveld FC, Smeets TJ, Daha MR, Kluin PM, Meinders AE, Maini RN. (1996). "Decrease in cellularity and expression of adhesion molecules by anti-tumor necrosis factor alpha monoclonal antibody treatment in patients with rheumatoid arthritis. ." Arthritis Rheum **39**(1077-1081).

ten Hove T, van Montfrans C, Peppelenbosch MP, van Deventer SJH. (2002). "Infliximab treatment induces apoptosis of lamina propria T lymphocytes in Crohn’s disease." Gut **50**: 206-211.

Trzonkowski P, Bieniaszewska M, Juścińska J, Dobyszuk A, Krzystyniak A, Marek N, Myśliwska J, Hellmann A. (2009a). "First-in-man clinical results of the treatment of patients with graft versus host disease with human ex vivo expanded CD4+CD25+CD127- T regulatory cells." Clin Immunol. **133**(1): 22-26.

Trzonkowski P, Szaryńska M, Myśliwska J, Myśliwski A. (2009b). "Ex vivo expansion of CD4(+)CD25(+) T regulatory cells for immunosuppressive therapy." Cytometry A **75**(3): 175-188.

Trzonkowski P, Szmit E, Mysliwska J, Dobyszuk A, Mysliwski A. (2004). "CD4+CD25+ T regulatory cells inhibit cytotoxic activity of T CD8+ and NK lymphocytes in the direct cell-to-cell interaction." Clin Immunol. **112**: 258-267.

Trzonkowski P, Zaucha JM, Mysliwska J, Balon J, Szmit E, Halaburda K, Bieniaszewska M, Mlotkowska M, Hellmann A, Mysliwski A. (2004). "Differences in kinetics of donor lymphoid cells in response to G-CSF administration may affect the incidence and severity of acute GvHD in respective HLA-identical sibling recipients." Med Oncol. **21**(1): 81-94.

van den Brande JM, Braat H, van den Brink GR, et al. (2003). "Infliximab but not etanercept induces apoptosis in lamina propria T-lymphocytes from patients with Crohn's disease. ." Gastroenterology **124**(7): 1774-1785.

van den Brandt J, Fischer HJ, Walter L, Hünig T, Klöting I, Reichardt HM.(2010) "Type 1 diabetes in BioBreeding rats is critically linked to an imbalance between Th17 and regulatory T cells and an altered TCR repertoire." J Immunol. **185**(4): 2285-94.

Velthuis JH, Unger WW, Abreu JR, Duinkerken G, et al. (2010). "Simultaneous detection of circulating autoreactive CD8+ T-cells specific for different islet cell-associated epitopes using combinatorial MHC multimers." Diabetes **59**(7): 1721-30.

Wong M, Ziring D, Korin Y, Desai S, Kim S, Lin J, Gjertson D, Braun J, Reed E, Singh RR. (2008). "TNFalpha blockade in human diseases: mechanisms and future directions." Clin Immunol. **126**(2): 121-136.

Wu A, Hua H, Munson S, McDevitt H. (2002). "Tumor necrosis factor-alpha regulation of CD4+CD25+ T cell levels in NOD mice. ." Proc Natl Acad Sci USA **99**(12287-12292).

Yang Z, Zhou Z, Huang G, Ling H, Yan X, Peng J, Li X. (2007). "The CD4(+) regulatory T-cells is decreased in adults with latent autoimmune diabetes." Diabetes Res Clin Pract. **76**(1): 126-31.

You S, Slehoffer G, Barriot S, Bach JF, Chatenoud L. "Unique role of CD4+CD62L+ regulatory T cells in the control of autoimmune diabetes in T cell receptor transgenic mice." Proc Natl Acad Sci U S A. **101**( Suppl. 2): 14580-5.

Zavattari P, Deidda E, Pitzalis M, Zoa B, Moi L, Lampis R, Contu D, Motzo C, Frongia P, Angius E, Maioli M, Todd JA, Cucca F. (2004). "No association between variation of the FOXP3 gene and common type 1 diabetes in the Sardinian population." Diabetes **53**(7): 1911-4.

**Patient’s consent form**

1. Title:

# “Cellular Therapy of Type 1 Diabetes with ex vivo expanded CD4+CD25+CD127- T regulatory cells”

**Duration of the study: 36 months**

**2. Introduction**

You are invited to take a part in medical experiment. Before you decide about the participation, it is important that you understand the aim and the course of the experiment. Please, read carefully the information provided and, if you wish, discuss it with your child, spouse, family or people you trust. If there is anything you do not understand or you wish to receive additional information, please ask the named physician who gave you this questionary (contact information is also provided et the end of this form).

No experimental procedure will be undertaken before you read and sign this form. If you decide to take a part in this study and sign this form, a complete copy of this form will be given to you.

**3. The aim of this study**

Type 1 Diabetes is a disease which usually develops in children as a result of destruction of pancreas – internal organ producing insulin. No insulin in the body results in high levels of sugar glucose in blood. This is manifested as disease symptoms: impaired consciousness leading to complete lose of it called ‘coma’; excessive drinking and urination, weight loss. In a long term, diabetes is responsible for the damage of kidneys, eyes and heart. Current medicine is capable to find early symptoms suggesting the beginning of diabetes or increased risk of the onset. The level of so-called anti-islet antibodies and some features of our cells, so-called HLA antigens, can be used for such a purpose. Usually, those anti-islet antibodies and some specific forms of HLA antigens can be found in patients with diabetes.

Currently, it is known that the disease is triggered by cells called lymphocytes, which attack and kill pancreatic insulin-producing cells.. It is also known that the destruction of pancreas is facilitated by the lack of some other cells, so-called T regulatory cells. T regulatory cells are able to stop lymphocytes from killing pancreatic cells but this effect requires high number of the former cells. Unfortunately, the number of T regulatory cells in blood is very low. It is estimated that one T regulatory cell can be found in a million of other blood cells and it is even more rare in patients with diabetes.

The experiment, you are invited to, aims at taking a sample of blood from your child and separating T regulatory cells from it. Then, the number of these cells will be increased in the laboratory. If the number of T regulatory cells will be increased sufficiently, we will administer them to your child as an accessory treatment of diabetes. Prior to the administration, the cells will be carefully checked for safety and quality and the named physician will consult you again about your decision on their infusion to your child.

This is experimental therapy which was previously used in our hospital in healthy volunteers and in some other disease. Up to now, we do not see any health problems is patients treated with these cells. Nevertheless, for the safety of your child, you and the child will be under special medical care during the blood drawing and infusion of T regulatory cells. We will also ask you to visit the hospital two weeks later, every two months for the first half a year, then every 3 months for the duration of the study (2 years) in order to assess the health status of your child. Whenever possible, these visits will be together with normal visits in outpatient clinic. During this visits after T regulatory cells infusion, we will draw a small amount of blood (15ml – volume of two spoons) in order to perform necessary laboratory tests.

**4. Programme of the experiment**

The experiment will be performer with patients treated for recently diagnosed type 1 diabetes in the Department of Pediatrics, Hematology and Oncology Medical University of Gdańsk. Lood will be drawn from patients in the amount adequate for age, body weight and health status. The volume of blood which can be drawn will be calculated according to the following equation: Maximal volume of blood to be drawn is equal to 1% of the body weight but no more than 250ml [e.g. child weighting 20kg – 200ml of blood is allowed; child weighting 40kg – 250ml of blood is allowed]. When necessary, the blood will be drawn in sedation with anaesthesiological assistance. The blood will be then prepared in the Blood Bank in order to separate plasma, white cells and red cells. The Plasma and white cells will be used in the study and erythrocytes will serve as a back up and can be transfused back if necessary. White cells will be used to separate T regulatory cells which will be then cultured under safe conditions in order to increase their number. If successful, the cells will be checked for their safety and quality (in microbiology and immunology laboratories) and administered back to the patient.

We expect that this procedure will be sufficient to stop or at least delay the progress of diabetes.

**5. Benefits from participation**

Your child will receive therapy which may stop or delay the onset of diabetes.

**6. Cessation of participation**

At any time, you are allowed to stop your participation in the study. Your wish will be fully respected and will not affect routine treatment of your child.

**7. Regulations and Data protection**

All procedures of this experiment are concordant with ‘the Medical Experiment requirements’ chapter of the Medical Doctor Act (Parliament of the Republic of Poland 1999 with amendments). All information obtained by the investigators during this study are guarded by the medical secret. All information on you and your family will be only use for the purpose of this study and will be not used in any other purpose. In particular, the information will not be shared with other people and institution unless your written consent is given for that. The exception for that are representatives of the approved government agencies, the Ethics Committee, and other regulatory agencies when approval is given by appropriate court.

There is an option for blood storing for future analysis in this study. It will be only performed if you agree in separate document.

**8. Contact**

If there is anything you do not understand or you wish to receive additional information, please ask the physician who gave you this questionary. This is the named physician responsible for your participation in this study.

Name....................................................................phone...............................................

**Patient’s consent form**

Title: “Cellular Therapy of Type 1 Diabetes with ex vivo expanded CD4+CD25+CD127- T regulatory cells”

**I attest that I have read and understand all information in this form and I have got possibility to ask the questions and received satisfactory responses.**

**I agree to take a part in this study. I understand that I will receive signed and dated copy of this form which can be kept for my files.**

**Parent’s signature Date Name and surname of the patient in printed capital letters)**

**Patient’s signature Date Name and surname of the**

**(if more than 16 yo) patient in printed capital letters)**

**Physician’s signature Date Name and surname of the physician in printed capital letters)**

**If the person invited to take a part in this study is unable to read this form, two eye-witness who assisted the person in reading must sign.**

**Eye-witness’ signature Date Name and surname of the Eye-witness in printed capital letters)**

**Eye-witness’ signature Date Name and surname of the Eye-witness in printed capital letters)**

**
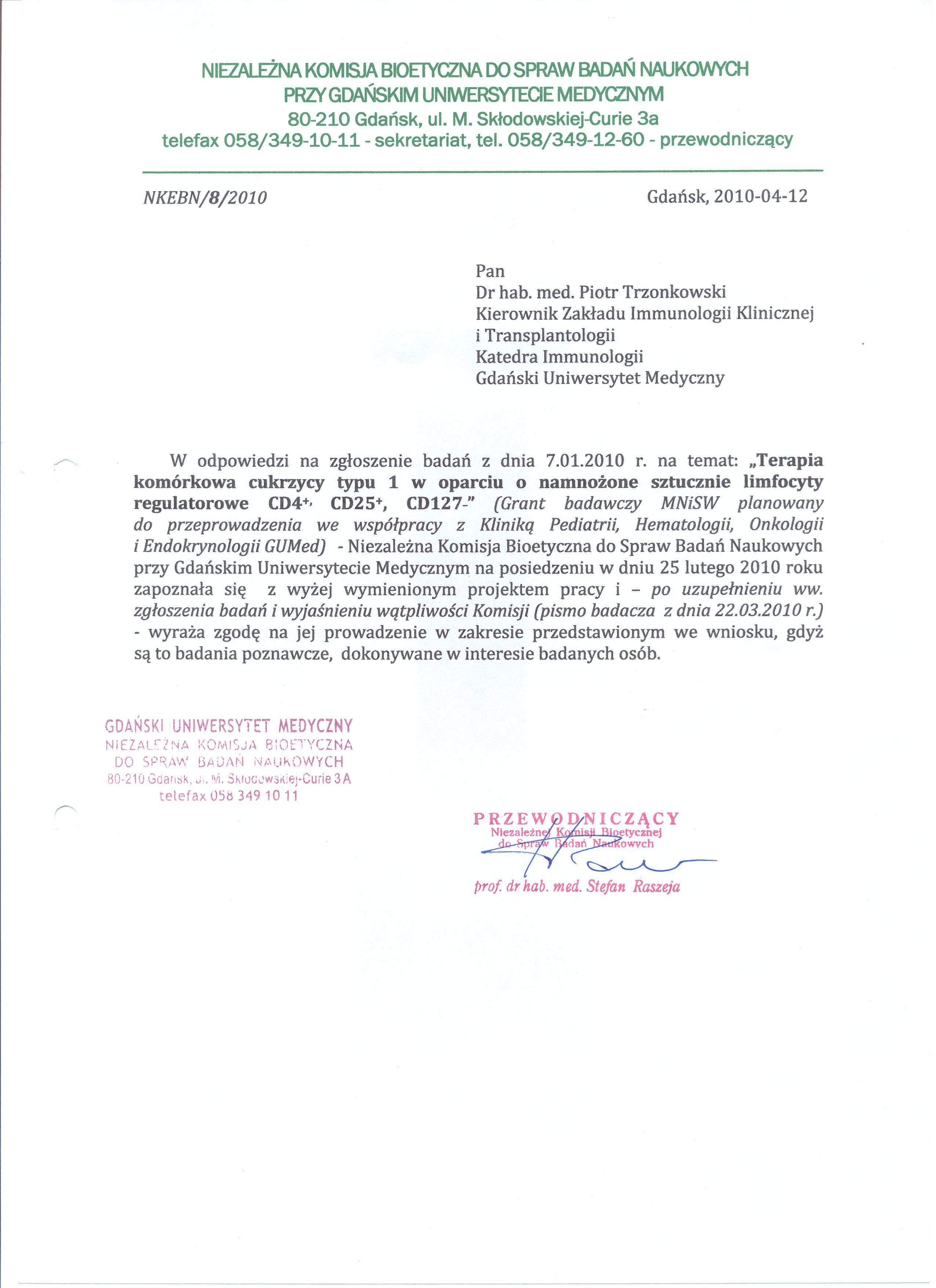
**
