# Supplementary material for: Factors affecting long-term efficacy of T regulatory cell-based therapy in type 1 diabetes
Source: J Transl Med. 2016 Dec 1;14:332. doi: 10.1186/s12967-016-1090-7 (PMC5131539; doi:10.1186/s12967-016-1090-7)
Supplement: Supplementary file 2 — Additional file 2. Statistics. [file 12967_2016_1090_MOESM2_ESM.docx]

## Factors affecting long-term efficacy of T regulatory cell-based therapy in type 1 diabetes

**Supplement 1**

Abbreviations:

Anti-GAD65 - glutamic acid decarboxylase autoantibodies

Anti-ZnT8 - zinc transporter 8 autoantibodies

7-AAD - Aminoactinomycin D

BAFF – B cell activating factor

b.w. – body weight

FACS – fluorescence activated cell sorter

GMP – good manufacturing practice

IFNγ - interferon γ

IL – interleukin

IAA – insulin autoantibodies

IA2 – islet antigen 2 antibodies

KW - Kruskal-Wallis ANOVA

MMTT – mixed meal tolerance test

MW – U-Mann-Whitney test

T1DM – type 1 diabetes

Tcm - T central memory

Teff – T effector/conventional cells

Tem - T effector memory

Tn – T naïve

Tregs – T regulatory cells

TNFα – Tumour necrosis factor α

VEGF – vascular endothelial growth factor

vs. – versus

## Beta-cell function [Figure 1]

**Beta-cell function was the best preserved in those treated with two doses of Tregs, slightly worse in those treated with one dose and the worst in non-treated controls.**

The effect measured as fasting C-peptide was significantly better in those treated with single or two doses of Tregs as compared to non-treated controls throughout the study.

Kruskal-Wallis ANOVA – comparison for all three groups:

time 0: Chi^2^ = 3,43; p = 0,180

4 months: Chi^2^ = 8,57; p = 0,014

1 year: Chi^2^ = 10,15; p = 0,006

2 years: Chi^2^ = 3,46; p = 0,177

Nevertheless, this difference became insignificant at 2 years post-recruitment between single dose group and non-treated controls (U-Mann Whitney test Z=0.63 p=0.43), while was still significant when two doses vs. non-treated controls were compared (U-Mann Whitney test z=2.04 p=0.04).

The difference was also seen when insulin administration was analyzed. Both groups treated with Tregs required lower doses of insulin throughout the studies. In the group treated with two doses two patients were insulin independent more than one year after infusion and all but one required less than 0.5 UI of insulin per kg b.w.

Kruskal-Wallis ANOVA – comparison for all three groups:

time 0: Chi^2^ = 5,21; p = 0,074

4 months: Chi^2^ = 8,351; p = 0,015

1 year: Chi^2^ = 7,86; p = 0,020

2 years: Chi^2^ = 5,88; p = 0,052

Importantly, the doses were adjusted to the markers of metabolic control such as Hb1C and fasting glucose and although the group treated with two doses required the lowest levels of exogenous insulin, these parameters were the best controlled, notably at two years post inclusion.

Kruskal-Wallis ANOVA for HbA1c – comparison for all three groups:

time 0: Chi^2^ = 8,06; p = 0,018

4 months: Chi^2^ = 0,78; p = 0,676

1 year: Chi^2^ = 7,13; p = 0,028

2 years: Chi^2^ = 3,233; p = 0,197

Kruskal-Wallis ANOVA for fasting glucose – comparison for all three groups:

time 0: Chi^2^ = 2,27; p = 0,322

4 months: Chi^2^ = 6,95; p = 0,031

1 year: Chi^2^ = 0,89; p = 0,639

2 years: Chi^2^ = 6,79; p = 0,034

The better function of the islets in the groups treated with Tregs was also confirmed with mixed meal tolerance test MMTT at 2 years post inclusion [Figure 2]. Both treated groups were characterized by better stimulated c-peptide profiles than those non-treated. The difference between the groups treated with 2 doses and those non-treated was significant. Similar comparison between those treated with one dose and those untreated was insignificant.

Kruskal-Wallis ANOVA – comparison for all three groups:

0-fasting: Chi^2^ = 3,93; p = 0,139

15': Chi^2^ = 3,88; p = 0,143

30': Chi^2^ = 5,40; p = 0,067

60': Chi^2^ = 5,92; p = 0,051

90': Chi^2^ = 4,90; p = 0,085

120': Chi^2^ = 4,90; p = 0,085

150': Chi^2^ = 5,27; p = 0,071

180': Chi^2^ = 4,90; p = 0,085

210': Chi^2^ = 5,32; p = 0,069

240': Chi^2^ = 4,27; p = 0,118

Kruskal-Wallis ANOVA – comparison One dose vs. NO Tregs:

0-fasting: Chi^2^ = 0; p = 1.0

15': Chi^2^ = 0,06; p = 0,792

30': Chi^2^ = 0,06; p = 0,792

60': Chi^2^ = 0; p = 1.0

90': Chi^2^ = 0,06; p = 0,792

120': Chi^2^ = 0,06; p = 0,792

150': Chi^2^ = 0,03; p = 0,843

180': Chi^2^ = 0,06; p = 0,792

210': Chi^2^ = 0,06; p = 0,792

240': Chi^2^ = 0,07; p = 0,790

Kruskal-Wallis ANOVA – comparison Two doses vs. NO Tregs:

0-fasting: Chi^2^ = 4,42; p = 0,035

15': Chi^2^ = 4,39; p = 0,036

30': Chi^2^ = 6,51; p = 0,010

60': Chi^2^ = 7,11; p = 0,007

90': Chi^2^ = 5,94; p = 0,014

120': Chi^2^ = 5,94; p = 0,014

150': Chi^2^ = 6,51; p = 0,010

180': Chi^2^ = 5,94; p = 0,014

210': Chi^2^ = 6,50; p = 0,010

240': Chi^2^ = 5,42; p = 0,019

## Tregs levels in vivo [Figure 3]

The level of Tregs increased every time, the cells were administered. Nevertheless, increased level of Tregs was not sustained and decreased to the baseline at 2 years after commencing the trial. In addition, the increase in the level of Tregs after the second administration was not as high as after the first administrations.

Kruskal-Wallis ANOVA for CD3+CD4+FoxP3+ Tregs, comparison for all three groups:

time 0: Chi^2^ = 12,83; p = 0,002

4 months: Chi^2^ = 5,98; p = 0,050

1 year: Chi^2^ = 9,98; p = 0,006

2 years: Chi^2^ = 0,379; p = 0,827

There was significant correlation between the percentages of the two phenotypes of Tregs CD3+CD4+FoxP3+ versus CD3+CD4+CD25highCD127- (Spearman for all patients R=0.33 p=0.01, non-treated R=0.30 p=0.09; single dose R=0.22 p=0.17; R=0.39 p=0.002). Nevertheless, the analysis of the content of CD25highCD127- gate revealed that with the time of diabetes progression the percentage of FoxP3+ cells was decreasing in the gate (Wilcoxon test: day 0 vs 24months: NO Tregs Z=2.053, p=0.04; Single dose Z=2.086, p=0.03; Two doses Z=1.35, p=0.17). It was reversible in patients after the second dose of Tregs. The percentage of FoxP3+ cells in the CD25highCD127- gate was significantly highest in the group treated with two doses of Tregs among all the three groups analyzed at 24months (Kruskal Wallis ANOVA Chi^2^=6.66 p=0.009).

## Tregs subsets in vivo [Figure 4]

The analysis of Tregs subsets may give some insight into the dynamics of the cells infused to the patients. The percentage of CD62L+CD45RA- T central memory Tregs (Tcm) was increasing after administration of Tregs. There was a significant swap from CD62L+CD45RA+ naïve (Tn) phenotype to CD62L+CD45RA- Tcm phenotype of Tregs after first infusion in both groups and less significant swap after the second infusion.

(Wilcoxon test day-10 vs. day0: **Tn Tregs:** Single dose Z=2.002, p=0.031; Two doses Z=2.92, p=0.003; 6m vs. 9m in Two doses: Z=1.20 p=0.23; **Tcm Tregs:** Single dose Z=1.32, p=0.18; Two doses Z=2.92, p=0.003; 6m vs. 9m in Two doses: Z=0,33 p=0.93; **Tcm/Tn Tregs ratio:** Single dose Z=1.32, p=0.18; Two doses Z=3.01, p=0.002; 6m vs. 9m in Two doses: Z=0.17 p=0.87).

## Immune markers of disease progression

[Figure 4]

Control non-treated group was characterized by significant swap of Tregs subsets throughout the follow-up. As compared to the baseline, there was an increase in the level of Tcm Tregs at the expense of of naïve Tregs at 24months . Similar, but only partially significant, changes were seen in patients treated with single dose of Tregs. These differences were the least seen in the group treated with two doses of Tregs.

(Wilcoxon test day-10 vs 24m **Tcm/Tn ratio Tregs:** NO Tregs Z=6.86, p=0.021, Single dose Z=2.89, p=0.088; Two doses Z=1.88, p=0.16).

(Wilcoxon test day-10 vs 24 **Tn Tregs:** NO Tregs Z=2.29, p=0.021, Single dose Z=1.52, p=0.120; Two doses Z=0.50, p=0.61).

(Wilcoxon test day-10 vs 24m **Tcm Tregs:** NO Tregs Z=2.42, p=0.011, Single dose Z=2.21, p=0.020; Two doses Z=0.85, p=0.39).

Among all the groups, the patients treated with two doses of Tregs were characterized by the most preserved (unchanged) phenotype - with the highest level of Tn Tregs and the lowest level of Tem Tregs – at 24months post inclusion.

Kruskal-Wallis ANOVA – comparison of the percentage of Tn, Tcm, Tem and Tcm/Tn ratio of Tregs between all three groups of patients at 24months post inclusion

Tcm/Tn Chi^2^ = 7,20 df = 2 p = 0,027

Tn Chi^2^ = 6,20 df = 2 p = 0,045

Tcm Chi^2^ = 7,20 df = 2 p = 0,027

Tem Chi^2^ = 3,76 df = 2 p = 0,152

## [Figure 5]

Among the cytokines measured from sera of the patients, proinflammatory ones revealed some pattern of concentrations with the time. Serum levels of IL6 were consistently increasing with the time, regardless of the therapy (there was a slight and transient decrease after the first dose in the group administered with two doses of Tregs). All patients followed revealed higher levels of IL6 at 24 months after inclusion

(Wilcoxon test day0 vs 24m: Single dose Z=1.66, p=0.020; Two doses Z=2.5, p=0.001).

In the case of TNFα and IL1, each infusion of Tregs in both groups was associated with temporary decrease in the level of these cytokines.

(Wilcoxon test

**TNFα** day-10 vs day0: Single dose Z=2.95, p=0.033; Two doses Z=2.67, p=0.046; 6m vs. 9m in Two doses: Z=0.34 p=0.67;

**IL1** day-10 vs day0: Single dose Z=1.16, p=0.09; Two doses Z=1.48, p=0.013; 6m vs. 9m in Two doses: Z=0.34 p=0.67).

As compared to the baseline, the level of all these cytokines was increased in the control non-treated group at 12months. It was also true for IL6 in the treated patients

(Wilcoxon test day0 vs 12m: TNFα Z=1.50, p=0.076; IL1 Z=1.82, p=0.022; IL6: Z=1.93 p=0.012).

Apart from IL2 levels, no significant changes were found in the levels of other cytokines measured.

## [Figure 6]

There was a correlation between the levels of CD3^+^CD4^+^FoxP3^+^ Tregs in vivo and IA2, when all patients were taken into analysis (Spearman’s rank correlation R=-0.304, p=0.05).

Figure 1S: The correlation between the levels of CD3^+^CD4^+^FoxP3^+^ Tregs in vivo and IA2, when all patients were taken into analysis.

There were no other correlations between T1DM autoantibodies (anti-GAD65, IAA, IA2, anti-ZnT8) and Tregs levels or the dose of Tregs administered or the cytokines assessed.

## Neither between-group comparison or the analysis of the dynamics of autoantibodies level throughout the follow revealed significant differences.

## Tregs and IL2 [Figure 7]

A sustainability of Tregs in the body after infusion can be linked with IL2 levels. This is the cytokine known to influence survival and function of Tregs and it was also used in high concentrations to expand Tregs for clinical application in the study.

In the first set of in vitro experiments, a sample of Tregs from the preparation used in the treatment was further cultured in vitro with different concentrations of IL2 and cell survival was measured. While no supplementation of IL2 was inevitably associated with rapid decrease in cell survival, relatively low concentrations of IL2 starting from 10UI/ml were enough to limit cell death. IL2 should be present in the culture constantly as delayed adding back of the cytokine had only minor effect on the survival.

Kruskal-Wallis ANOVA – comparison of survival of Tregs in four IL2 concentrations: 0, 10, 100 and, 1000 UI/ml

day1: Chi^2^ = 4,47; p = 0,214

day2: Chi^2^ = 11,75; p = 0,008

day3: Chi^2^ = 10,34; p = 0,015

day4: Chi^2^ = 10,66; p = 0,013

day5: Chi^2^ = 8,24; p = 0,030

day6: Chi^2^ = 6,20; p = 0,102

day7: Chi^2^ = 4,65; p = 0,198

day8: Chi^2^ = 1,16; p = 0,761

All significant difference were between 0 UI/ml and all others

Interestingly, as compared to autologous sentinel T effectors, Tregs were much more sensitive to deprivation of IL2.

Kruskal-Wallis ANOVA – comparison of survival of Tregs vs. T effectors in 0 UI/ml of IL2

day1: Chi^2^ = 0.11 p = 0.970

day2: Chi^2^ = 0.72; p = 0.450

day3: Chi^2^ = 6,86; p = 0,008

day4: Chi^2^ = 6,81; p = 0,009

day5: Chi^2^ = 6,81; p = 0,009

day6: Chi^2^ = 6,20; p = 0,012

day7: Chi^2^ = 5,77; p = 0,016

day8: Chi^2^ = 0,60; p = 0,438

This might be related to some specific features of T effectors, such as the capabilities of autocrine production of IL2, which enabled them to survive in vitro. As Tregs coexist in the body with effectors, coculture of these two populations was performed to mimic in vivo conditions. Simple coculture of Tregs with Teffectors improved viability, which was significantly synergized when exogenous IL2 was also added.

Kruskal-Wallis ANOVA – comparison of survival of Tregs alone vs. cocultures of Tregs with T effectors in 0 UI/ml of IL2

day1: Chi^2^ = 0,60; p = 0,438

day2: Chi^2^ = 5,14; p = 0,023

day3: Chi^2^ = 3,75; p = 0,052

day4: Chi^2^ = 7,0; p = 0,008

day5: Chi^2^ = 2,4; p = 0,121

day6: Chi^2^ = 2,28; p = 0,130

day7: Chi^2^ = 0,60; p = 0,438

day8: Chi^2^ = 0,33; p = 0,340

Again, the effect was seen starting from the cocultures with the lowest concentrations of IL2 (10 UI/ml) up to the highest ones.

Kruskal-Wallis ANOVA – comparison of survival in cocultures of Tregs with T effectors in four IL2 concentrations: 0, 10, 100 and, 1000 UI/ml

day1: Chi^2^ = 1,16; p = 0,761

day2: Chi^2^ = 5,38; p = 0,145

day3: Chi^2^ = 2,83; p = 0,418

day4: Chi^2^ = 4,66; p = 0,197

day5: Chi^2^ = 5,62; p = 0,013

day6: Chi^2^ = 2,28; p = 0,130

day7: Chi^2^ = 9,15; p = 0,027

day8: Chi^2^ = 6,00; p = 0,111

Dependency of Tregs from IL2 found in vitro was further confirmed with the levels of this cytokine in sera of the patients. Shortly after Tregs infusions, notably in the group treated with two doses of Tregs, the concentrations of IL2 were transiently decreasing, possibly utilized by infused Tregs.

(Wilcoxon test day-10 vs day0: Single dose Z=0.50, p=0.61; Two doses Z=0.67, p=0.50; 6m vs. 9m in Two doses: Z=2.01 p=0.04).
